# ChemBioSim: Enhancing Conformal Prediction of In Vivo Toxicity by Use of Predicted Bioactivities

**DOI:** 10.1021/acs.jcim.1c00451

**Published:** 2021-06-21

**Authors:** Marina Garcia de Lomana, Andrea Morger, Ulf Norinder, Roland Buesen, Robert Landsiedel, Andrea Volkamer, Johannes Kirchmair, Miriam Mathea

**Affiliations:** † BASF SE, Ludwigshafen am Rhein 67063, Germany; ‡ Department of Pharmaceutical Sciences, Faculty of Life Sciences, University of Vienna, Vienna 1090, Austria; § In Silico Toxicology and Structural Bioinformatics, Institute of Physiology, Charité Universitätsmedizin Berlin, Charitéplatz 1, Berlin 10117, Germany; ∥ MTM Research Centre, School of Science and Technology, Örebro University, Örebro SE-70182, Sweden

## Abstract

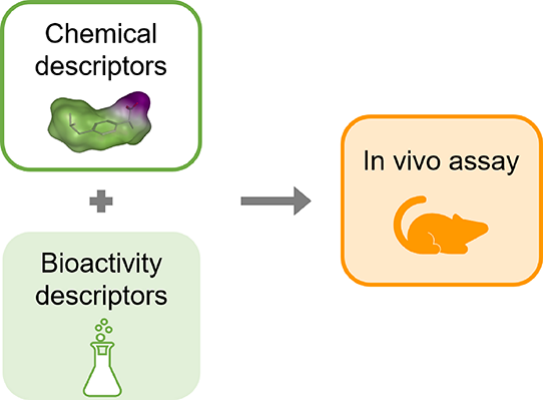

Computational methods such as machine learning approaches have a strong track record of success in predicting the outcomes of in vitro assays. In contrast, their ability to predict in vivo endpoints is more limited due to the high number of parameters and processes that may influence the outcome. Recent studies have shown that the combination of chemical and biological data can yield better models for in vivo endpoints. The ChemBioSim approach presented in this work aims to enhance the performance of conformal prediction models for in vivo endpoints by combining chemical information with (predicted) bioactivity assay outcomes. Three in vivo toxicological endpoints, capturing genotoxic (MNT), hepatic (DILI), and cardiological (DICC) issues, were selected for this study due to their high relevance for the registration and authorization of new compounds. Since the sparsity of available biological assay data is challenging for predictive modeling, predicted bioactivity descriptors were introduced instead. Thus, a machine learning model for each of the 373 collected biological assays was trained and applied on the compounds of the in vivo toxicity data sets. Besides the chemical descriptors (molecular fingerprints and physicochemical properties), these predicted bioactivities served as descriptors for the models of the three in vivo endpoints. For this study, a workflow based on a conformal prediction framework (a method for confidence estimation) built on random forest models was developed. Furthermore, the most relevant chemical and bioactivity descriptors for each in vivo endpoint were preselected with lasso models. The incorporation of bioactivity descriptors increased the mean F1 scores of the MNT model from 0.61 to 0.70 and for the DICC model from 0.72 to 0.82 while the mean efficiencies increased by roughly 0.10 for both endpoints. In contrast, for the DILI endpoint, no significant improvement in model performance was observed. Besides pure performance improvements, an analysis of the most important bioactivity features allowed detection of novel and less intuitive relationships between the predicted biological assay outcomes used as descriptors and the in vivo endpoints. This study presents how the prediction of in vivo toxicity endpoints can be improved by the incorporation of biological information—which is not necessarily captured by chemical descriptors—in an automated workflow without the need for adding experimental workload for the generation of bioactivity descriptors as predicted outcomes of bioactivity assays were utilized. All bioactivity CP models for deriving the predicted bioactivities, as well as the in vivo toxicity CP models, can be freely downloaded from https://doi.org/10.5281/zenodo.4761225.

## Introduction

Modern toxicity testing heavily relies on animal models, which entails ethical concerns, substantial costs, and difficulties in the extrapolation of results to humans.^1^ The increasing amount and diversity of not only drugs but also more generally of chemicals present in the environment and the lack of knowledge about their toxic potential require the development of more efficient toxicity assessment tools.

In recent years, in silico tools for toxicity prediction have evolved into powerful methods that can help to decrease animal testing.^2−4^ This is particularly true when applied in tandem with in vitro methods.^5^ Machine learning (ML) models trained on data sets of compounds with known activities for an assay can be used as predictive tools for untested compounds.^6^ These models are generally trained on chemical and structural features of compounds with measured activity values.^7^ However, the outcomes of in vivo toxicological assays depend on a number of biological interactions such as the administration, distribution, metabolism, and excretion (ADME) and the interaction with different cell types.^4^ The ability of chemical property descriptors to capture these complex interactions and, consequently, the predictive power of ML models trained on these molecular representations are limited. By the example of classification models for hit expansion^8,9^ and toxicity prediction,^10−13^ recent studies have shown that the predictive power of in silico models can be improved by the amalgamation of chemical and biological information. More specifically, it has been shown that bioactivity descriptors could help to infer the activity of new substances by capturing the similarity of compounds in the biological space, i.e., identifying those compounds that behave similarly in biological systems (but may be chemically dissimilar). However, options to integrate biological data into models are limited by the sparsity of the available experimental data. In principle, the use of bioactivity features in ML requires compounds of interest to be tested in all assays conforming the bioactivity descriptor set. Norinder et al.^14^ however showed, by the example of conformal prediction (CP) frameworks built on random forest (RF) models, that the use of predicted bioactivity descriptors in combination with chemical descriptors can yield superior cytotoxicity and bioactivity predictions while circumventing the problems of sparsity of data and extensive testing. CP models are a robust type of confidence predictors that generate predictions with a fixed error rate determined by the user.^15^ To estimate the confidence of new predictions, the predicted probabilities of a set of compounds with known activity (calibration set) are used to rank the predicted probabilities for new compounds and calculate their so-called *p*-values (i.e., calibrated probabilities). An additional feature of CP models is their ability to handle data imbalance and predict minority classes more accurately.^16^

The CP approach offers the advantage of a mathematical definition of a model’s applicability domain (AD); i.e., chemical space within the model makes predictions with a defined reliability based on the allowed error rate.^17^ Other common approaches for defining the applicability domain are based on compound similarity or predicted probability and a more or less arbitrary (user-defined) threshold. However, CP models return a statistically robust class membership probability for each class. Under the exchangeability assumption of the samples (assumption also made for classical ML models), the observed error rate returned by CP models will be equal to (or very close to) the allowed (i.e., user-defined) error rate.

The aim of this study is to determine if, and to what extent, classification models for the prediction of in vivo toxicity endpoints can benefit from integrating chemical representations with data from biological assays. To include the biological assay information in the models, predicted bioactivities were derived from 373 CP models, each representing an individual biological assay. The results obtained for models trained exclusively on chemical descriptors (“CHEM”), trained exclusively on bioactivity (“BIO”) descriptors, or trained on the combination of chemical and bioactivity descriptors (“CHEMBIO”) were analyzed for three toxicological in vivo endpoints: in vivo genotoxicity (with the in vivo micronucleus test (MNT)), drug-induced liver injury (DILI), and cardiological complications (DICC).

The in vivo MNT assay is used to detect genetic (clastogenic and aneugenic) damage induced by a substance causing the appearance of micronuclei in erythrocytes or reticulocytes of mice or rats.^18^ DILI describes the potential hepatotoxicity of a compound. Although there is no consensus method for assessing the DILI potential of a compound, the U.S. Food and Drug Administration (FDA) proposed a systematic classification scheme based on the FDA-approved drug labeling.^19^ The DICC endpoint comprises five cardiological complications induced by drugs and annotated in clinical reports: hypertension, arrhythmia, heart block, cardiac failure, and myocardial infarction.

Severe organ toxicity, as observed with DILI and DICC, but also genotoxicity (which can lead to carcinogenesis and teratogenic effects) must be avoided and hence recognized early in the development of industrial chemicals and drugs. Both hepatic and cardiovascular adverse effects are listed as two of the most common safety reasons for drug withdrawals^20^ and failures in drug development phases I–III.^21^ Moreover, REACH, the chemical control regulation in the European Union, is requiring the in vivo MNT as follow up of a positive result in any genotoxicity test in vitro.^22^ The Organisation for Economic Co-operation and Development (OECD) Guideline 474 and the International Council for Harmonisation of Technical Requirements for Pharmaceuticals for Human Use (ICH) list the in vivo MNT assay as one of the recommended tests for detecting genotoxicity, as it can account for ADME factors and DNA repair processes.^18,23^

This study introduces an improvement of the in silico prediction of in vivo toxicity endpoints by considering the activity of compounds in multiple biological test systems. We show that predicted bioactivities, which present the benefit of not needing further experimental testing for new compounds, are often enough to achieve ML models with increased performance.

## Materials and Methods

### Data Sets

In the following paragraphs, the data from biological assays used for generating descriptors based on predicted bioactivities are introduced followed by the data related to the three in vivo toxicological endpoints (MNT, DILI, and DICC). Finally, the reference data sets used to analyze the chemical space covered by the in vivo endpoints are described.

All information required for the download of any of the data sets used for modeling in this study (including download links, exact json queries, as well as MD5 file checksums) are provided in Table S1 (for the in vivo endpoints) and Table S2 (for the biological assays).

#### Biological Assays

For the generation of descriptors from predicted bioactivities, a total of 373 data sets (each belonging to a single biological assay) were collected (Table 1): 372 data sets from in vitro assays obtained from the ToxCast,^24^ eMolTox,^25^ and eChemPortal^26^ databases and the literature, and one data set from an in vivo assay (a human oral bioavailability assay) obtained from Falcón-Cano et al.^27^ From the ToxCast and eMolTox databases, only endpoints with at least 200 active and 200 inactive compounds listed (after structure preparation and deduplication; see the section Structure Preparation for details) were considered for modeling. Besides the endpoints selected from these two databases, data sets for assays covering genotoxicity, bioavailability, permeability, thyroid hormone homeostasis disruption, and P-glycoprotein inhibition were considered (Table 1). A more detailed description of the data collection and activity labeling of these data sets is provided in Table S2. The numbers of active and inactive compounds in each of the 373 data sets (after the structure preparation and deduplication steps) are reported in Table S3.

**Table 1:** Overview of Collected Assay Data

database/endpoint	description	source
ToxCast database	• 222 high-throughput screening assays, including endpoints related to cell cycle and morphology control, steroid hormone homeostasis, DNA-binding proteins, and other protein families (e.g., kinases, cytochromes, and transporters)	ToxCast database version 3.3^24^
eMolTox database	• 136 in vitro assays, including endpoints related to mutagenicity, cytotoxicity, hormone homeostasis, neurotransmitters, and several protein families (e.g., nuclear receptors, cytochromes, and cell surface receptors)	Ji et al.^25^
genotoxicity	• AMES mutagenicity assay	AMES assay: eChemPortal,^26^ Benigni et al.,^28^ Hansen et al.^29^
	• chromosome aberration (CA) assay	
	• mammalian mutagenicity (MM) assay	CA and MM assays: eChemPortal, Benigni et al.
bioavailability	• human oral bioavailability assay	Falcón-Cano et al.^27^
permeability	• Caco-2 assay	Wang et al.^30^
thyroid hormone homeostasis	• deiodinases 1, 2, and 3 inhibition assays	Garcia de Lomana et al.^31^
	• thyroid peroxidase inhibition assay	
	• sodium iodide symporter inhibition assay	
	• thyroid hormone receptor antagonism assay	
	• thyrotropin-releasing hormone receptor antagonism assay	
	• thyroid stimulating hormone receptor agonism and antagonism assays	
P-glycoprotein inhibition	• P-glycoprotein (ABCB1) inhibition assay	Broccatelli et al.^32^

#### In Vivo Endpoints

During the development of this study, a larger number of publicly available in vivo endpoint data sets were investigated for their suitability for modeling. Taking into account the quantity and quality of the data, as well as the regulatory relevance of the toxicological endpoints, three in vivo endpoints were selected for this study: MNT, DILI, and DICC. The collection of the respective data sets is introduced in the following paragraphs.

MNT Data Set. For the MNT assay, data from the European Chemicals Agency (ECHA) available at the eChemPortal were collected. Only experimental data derived according to the OECD Guideline 474 (or equivalent) were considered. All assay outcomes annotated as unreliable or related to compounds that are cytotoxic were discarded. All compounds (identified based on CAS numbers) with conflicting activity data were also removed. Additional data were obtained from the work of Benigni et al.,^28^ which includes curated data sets from the European Food Safety Authority (EFSA) data. In addition, data sets for MNT on mouse (1001 compounds) and rat (127 compounds) compiled by Yoo et al.^33^ and containing binary activity labels for MNT were obtained. These additional data sets include data, among other sources, from the FDA approval packages, the National Toxicology Program (NTP) studies, the U.S. EPA GENETOX database, the Chemical Carcinogenesis Information System (CCRIS) and the public literature. The mouse and rat data sets did not contain overlapping compounds and an overall MNT result (independent from the species) was derived for the 1128 compounds in the data set. The final data set (after the structure preparation and deduplication steps) contains a total of 1791 compounds (316 active and 1475 inactive compounds; Table 2).

**Table 2:** Overview of the Data Sets for the in Vivo Endpoints

	number of		
endpoint	active compounds	inactive compounds	ratio
MNT	316	1475	1:5
DILI	445	247	2:1
DICC	988	2268	1:2

DILI Data Set. The data for the DILI endpoint were obtained from the verified DILIrank data set compiled by the FDA.^34^ In this data set, drugs are classified as “Most-DILI-concern”, “Less-DILI-concern”, “No-DILI-concern”, and “Ambiguous-DILI-concern”. For the purpose of this study, compounds in the “Most-DILI-concern” and “Less-DILI-concern” classes were labeled as ″active″ and compounds in the “No-DILI-concern” class were labeled as ″inactive″. Compounds of the ″Ambiguous-DILI-concern″ class were removed from the data set. The final binary DILI data set contained 692 compounds (445 active and 247 inactive compounds).

DICC Data Set. For the DICC endpoint, the data set compiled by Cai et al.^35^ on different cardiological complications was used. In their work, Cai et al. gathered individual data sets for hypertension, arrhythmia, heart block, cardiac failure, and myocardial infarction from five databases: Comparative Toxicogenomics Database (CTD),^36^ SIDER^37^ (side effect resource), Offsides^38^ (database of drugs effects), MetaADEDB^39^ (adverse drug events database), and DrugBank.^40^ In this study, a unique DICC data set was built that combines the five data sets of Cai et al. In the DICC data set, compounds were labeled as “active” if they were measured to be active on at least one of the cardiological endpoints (and active, inactive, or “missing” on the remaining endpoints), and as “inactive” otherwise. This resulted in a data set of 3256 compounds after the structure preparation and deduplication steps (988 active and 2268 inactive compounds; see section Structure Preparation for details).

#### Reference Data Sets

Three reference data sets were obtained to represent the chemical space of pesticide active ingredients, cosmetic ingredients, and drugs in order to analyze the coverage of these types of substances by the in vivo endpoint data sets. The chemical space of pesticides was represented by the 2417 compounds (after structure preparation and deduplication; see the section Structure Preparation for details) collected in the Pesticide Chemical Search database^41^ (from the Environmental Protection Agency’s (EPA) Office of Pesticide Programs) and downloaded from the CompTox Dashboard.^42^ The chemical space of cosmetic ingredients was represented by the 4503 compounds (after structure preparation and deduplication) included in the COSMOS cosmetics database,^43^ created as part of a European Union project for determining the safety of cosmetics in industry without the use of animals, and downloaded from the CompTox Dashboard as well. The chemical space of drugs was represented by the 10087 (after structure preparation and deduplication) approved, experimental, or withdrawn drugs contained in DrugBank.^44^

### Structure Preparation

The structures of all molecules were prepared starting from the respective SMILES strings, which are directly available from most data resources. For resources that do not provide SMILES strings (e.g., eChemPortal and the work of Yoo et al.), this information was obtained by querying the PubChem PUG REST interface^45^ with the CAS numbers. CAS numbers for which no SMILES was retrieved by this PubChem search were queried with the NCI/CADD Chemical Identifier Resolver.^46^ For the 977 compounds that did not produce any match with this procedure either, the “RDKit from IUPAC” node of RDKit^47^ in KNIME^48^ was used in an attempt to derive a structure from the chemical name. For 131 out of these 977 compounds, the chemical structure was successfully derived with this method. The remaining 846 compounds, without known chemical structures (e.g., including compound mixtures and unspecific formulas), were removed.

All obtained SMILES notations were interpreted, processed, and standardized with the ChemAxon Standardizer^49^ node in KNIME. As part of this process, solvents and salts were removed, aromaticity was annotated, charges were neutralized, and structures were mesomerized (taking the canonical resonant form of the molecule). All compounds containing any element other than H, B, C, N, O, F, Si, P, S, Cl, Se, Br, and I were removed from the data set with the “RDKit Substructure Filter” node in KNIME. In the case of multicomponent compounds, the structures of the individual components forming the compound were compared. More specifically, the canonical SMILES of the components were derived with RDKit, and in case the components had identical canonical SMILES, one of them was kept; otherwise, the whole compound was filtered out. Lastly, compounds with fewer than four heavy atoms were discarded.

Canonical SMILES were derived with RDKit from all standardized compounds. For each endpoint data set, duplicate canonical SMILES with conflicting activity labels were removed from the respective endpoint data set.

A KNIME workflow with the specific steps and settings for the preparation of the structures as well as for the calculation of the chemical descriptors (see Descriptor Calculation section) is provided in the Supplementary Information.

### Descriptor Calculation

#### Chemical Descriptors

Molecular structures were encoded using count-based Morgan fingerprints with a radius of 2 bonds and a length of 2048 bytes, computed with the ″RDKit Count-Based Fingerprint″ node in KNIME. Morgan fingerprints encode circular environments and capture rather local properties of the molecules. To capture global molecular properties, all 119 1D and 2D physicochemical property descriptors implemented in the “RDKit Descriptor Calculation” node in KNIME were calculated. These descriptors encode properties such as the number of bonds and rings in a molecule, the number of particular types of atoms, or the polarity and solubility of the compound. Two acidic and two basic p*K*_a_ values were also calculated per molecule with the “p*K*_a_” KNIME node from ChemAxon.^50^ Missing p*K*_a_ values (for molecules without two acidic or basic groups) were replaced with the mean value of the data set.

#### Bioactivity Descriptors

**Figure 1:**
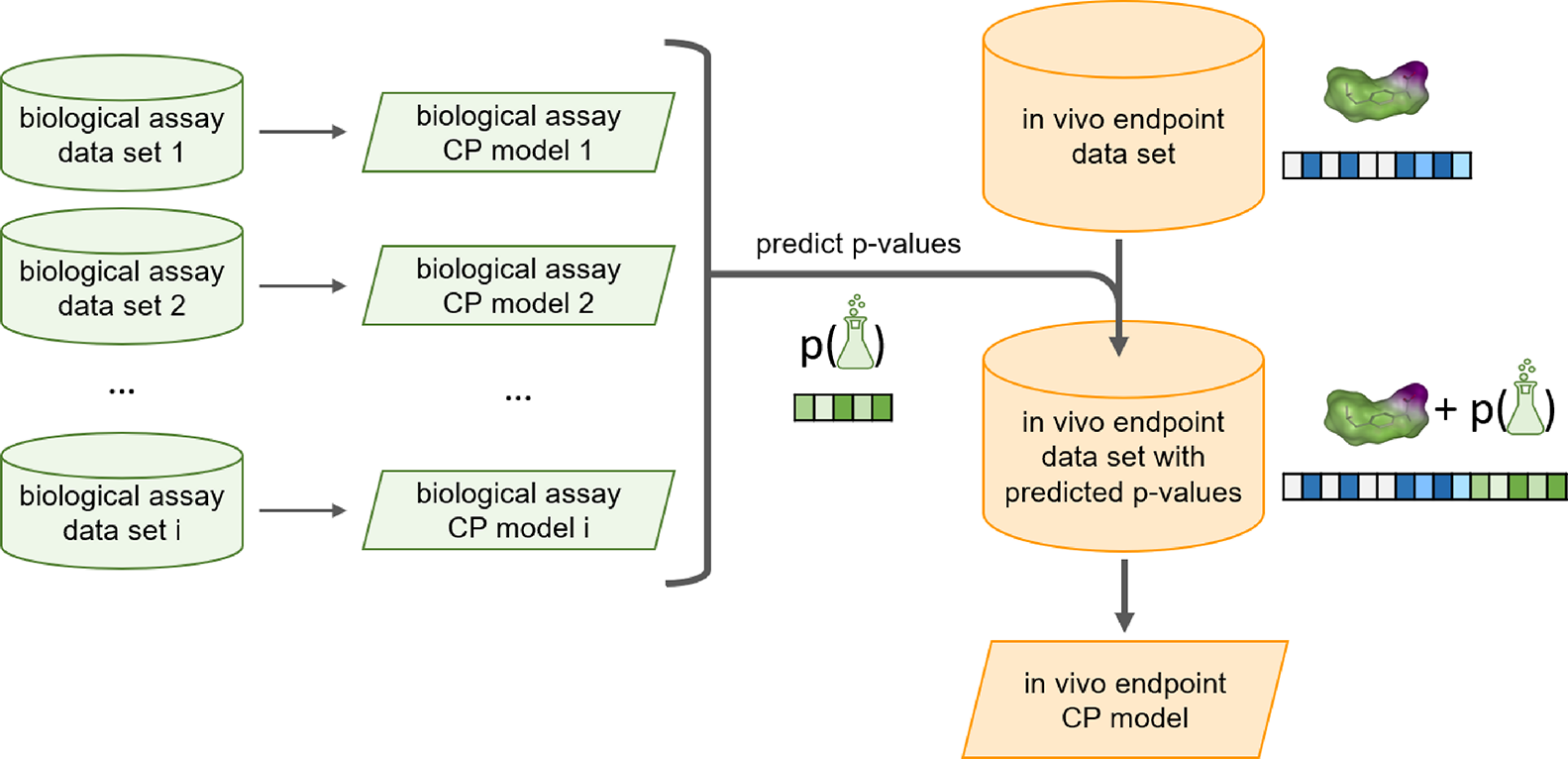
Workflow for the derivation of the bioactivity descriptors for the in vivo toxicity CP models. For each biological assay, a conformal prediction model is built and used to predict the *p*-values of the compounds in the three in vivo endpoint data sets. These predicted *p*-values are used as bioactivity descriptors, in combination with chemical descriptors, for training the models of the in vivo endpoints.

For the calculation of the bioactivity descriptors, first, 373 CP models—one per assay—were fitted on the respective biological assay sets (see the Data Sets section for details). The workflow for the generation of these models is explained in detail in the “Model development” section. With the generated bioactivity CP models, two *p*-values for each compound contained in the three in vivo endpoint data sets were predicted (Figure 1). Both the *p*-values for the active (p1) and for the inactive (p0) classes for each assay were used as bioactivity descriptors, resulting in 746 descriptors.

### Chemical Space Analysis

To visualize the chemical space covered by the data sets of the in vivo endpoints, dimensionality reduction was performed on a subset of 23 physically meaningful and interpretable molecular descriptors generated with RDKit (Table S4). For that purpose, the principal component analysis (PCA) implementation of scikit-learn^51^ was applied on the merged in vivo endpoint data sets (merged on the canonical SMILES). A further visualization of the chemical space defined by the complete CHEM and CHEMBIO descriptor sets was performed with the Uniform Manifold Approximation and Projection (UMAP).^52^ This method conducts a dimension reduction while maintaining the global structure of the data (i.e., the pairwise distance between samples). For each of the three in vivo endpoint data sets, a two-dimensional projection was performed on the CHEM and CHEMBIO descriptor sets, respectively, with 50 nearest neighbors, a minimum distance of 0.2, and use of the “euclidean” metric as the distance measure.

The molecular similarities of the compounds of the in vivo endpoint data sets and the collected pesticides, cosmetics, and drugs reference data sets were quantified with Tanimoto coefficients calculated from Morgan fingerprints with a radius of 2 bonds and a length of 1024 bits (fingerprints computed with the ″RDKit Fingerprint″ node in KNIME).

### Model Development for the Biological Assays and In Vivo Toxicity Endpoints

#### Workflow for the Development of CP Models

The same model development workflow was followed to train the CP models used for the calculation of the bioactivity descriptors, as well as to train the final models for the in vivo toxicity endpoints. Note that the structure preparation and chemical descriptor calculation was done in KNIME, but the following workflow was implemented in Python. All hyperparameters of the functions used in the workflow for deriving the CP models are specified in Table S5.

Prior to model development, a variance filter was applied to all features used as input for the in vivo toxicity CP models (including the bioactivity features if present) in order to remove any features with low information content. More specifically, any features with a variance (among the compounds in the respective data set) of less than 0.0015 were removed. Note that, in order to preserve the homogeneity of the input features, this variance filter was not part of the workflow for the biological assay CP model development (used to calculate the bioactivity descriptors). Also, in all cases (including the biological assay CP models), the features were scaled (by subtracting the mean and scaling to unit variance) prior to model development by applying the StandardScaler class of scikit-learn on each endpoint-specific data set.

**Figure 2:**
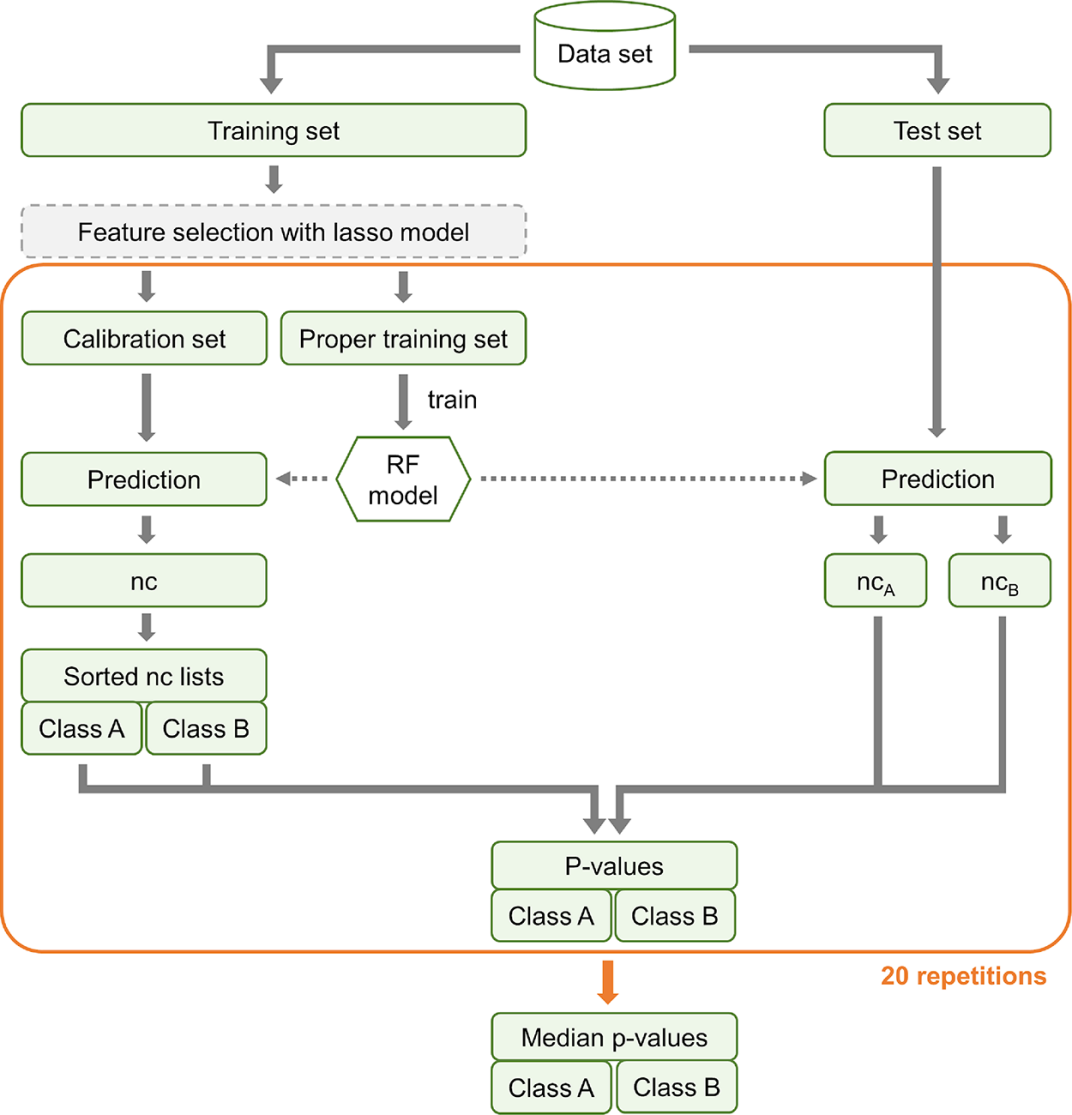
Workflow of the aggregated Mondrian CP set up for the development of the models for the biological assays and the in vivo endpoints. The aggregated CP framework included 20 random splits in calibration and proper training data sets, on which individual RF models were trained, and the resulting *p*-values per test compound were afterward averaged. The feature selection step was implemented with a lasso model and only included in the development of the in vivo toxicity CP models (in vivo toxicity CP models without feature selection were also trained for comparison).

For CP model development, each endpoint-specific data set was divided into 80% training and 20% test set using the StratifiedShuffleSplit class of scikit-learn (Figure 2). For performance assessment, this splitting of the data was performed within a 5-fold cross-validation (CV) framework. During each CV run, the training set was further divided (stratified) into a proper training set (70% of the training set) and a calibration set (30% of the training set) with the RandomSubSampler class from the nonconformist Python package.^53^ An RF model was trained on the proper training set using the scikit-learn implementation (with 500 estimators and default values for the rest of the hyperparameters). The trained RF model was then used to predict the probabilities of the compounds in the calibration set. From these probabilities, the so-called nonconformity score (nc score) was derived by applying a nonconformity error function, which yields low nc scores for predictions close to the true value. Here, the inverse probability error function from the nonconformist package (named “InverseProbabilityErrFunc”) was used to calculate the nc scores. This error function is defined as
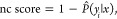
with *P̂*(*y_i_* | *x*) being the probability of predicting the correct class.

By definition, errors produced by CP models do not exceed the significance level ε (i.e., indicated error rate) under the assumption that training and test compounds are independent and belong to the same distribution. However, these errors may be unevenly distributed across classes. To achieve conditional validity with respect to the active and inactive classes, the Mondrian approach was used. Following the Mondrian CP approach, a sorted nc score list with the calculated nc scores of the calibration set was created for each class (active/inactive) independently. After calculating the nc scores (one per class) for the test compounds, their rank (with regard to the calibration set) in the respective list was calculated. The rank of the nc score of each test compound defines the predicted *p*-value for the respective class.

An aggregated CP approach^54^ was conducted by repeating the random splitting of the proper training and calibration sets 20 times. As a result, the *p*-values for a test set were calculated 20 times and the final *p*-value was derived from the median value.

CP models output a set of labels, which contain one class (“active” or “inactive”), both classes, or none. If the final *p*-value for any of the classes was higher than the significance level ε, the compound was assigned to that class (or to both classes if both *p*-values were higher than ε). Thus, based on the *p*-values and the significance level, the CP model determines whether a compound is within the applicability domain (AD) of the model.^55^ Compounds within the AD of the model are assigned to one or both classes and those outside of the AD are assigned to the empty class (i.e., no class label is assigned).

The predicted *p*-values obtained by applying the bioactivity CP models on the in vivo endpoint data sets (for the generation of the bioactivity descriptors) were used as is, and no class labeling was performed (i.e., no significance level was assigned). Instead, the *p*-values for both classes were considered.

#### In Vivo Toxicity CP Models Including Feature Selection

The workflow for developing the in vivo toxicity CP models that include feature selection is similar to the general workflow described in the previous section but additionally includes a least absolute shrinkage and selection operator (lasso) model.^56^ Lasso is a regression method that penalizes the coefficients of the input features for the selection of variables and the regularization of models. Some feature coefficients are shrunk to zero and therefore eliminated from the model.

In our workflow, a lasso model with the LassoCV implementation of scikit-learn was trained on the complete training set (prior to splitting the complete training set into proper training and calibration set; see Figure 2). To optimize the regularization parameter alpha of the lasso model, an inner 5-fold CV is applied. The list of coefficients assigned to each feature is obtained, and those features with a coefficient shrunken to zero are filtered out from the data set. Only the selected features (i.e., with a coefficient higher than zero) are used as input for the aggregated CP workflow described in the previous section.

In order to use the coefficients for ranking the features according to their importance for the analysis of the models, the mean among the absolute values of the coefficients obtained during each outer CV run was calculated.

Since the lasso model discards highly correlated features, considering only the lasso coefficients for the analysis of the most relevant features could lead to an underestimation of the importance of some biological assays. Therefore, this analysis was mainly based on the feature importance values of the RF models without feature preselection with lasso. The feature importance values of RF were extracted, and the mean across CV runs were calculated. Lastly, to better estimate the relative importance of each feature, a min-max normalization with the MinMaxScaler class of scikit-learn (with a range of 0.01 to one) was applied on the mean coefficients higher than zero and on the mean feature importance values of RF.

### Performance Evaluation of CP Models

Two important metrics for the evaluation of CP models were calculated based on all predictions of the respective test sets: the validity and the efficiency. CP models are proven to be valid (i.e., guarantee the error rate indicated by the user) if the training and test data are exchangeable.^15^ To achieve the indicated validity of the predictions, CP models output a set of class labels that can be empty, contain both labels, or only one of the labels (i.e., single class predictions). The validity is defined as the ratio of predictions containing the correct label (the “both” class set is therefore always correct and the “empty” set is always wrong). The efficiency measures the ratio of single class predictions (i.e., predictions containing only one class label) and, therefore, how predictive a model for a given endpoint is.

Additionally, the F1 score, Matthews correlation coefficient (MCC), specificity, sensitivity, and accuracy (both overall and independently for each class) were calculated (on the single class predictions only), to determine the model quality. The F1 score is the harmonic mean of precision and recall and is robust against data imbalance. The MCC considers all four classes of predictions (true positive, true negative, false positive, and false negative predictions) and takes values in the range of −1 to +1 (a value of +1 indicates perfect prediction). This metric is also robust against data imbalance. The specificity is determined by the proportion of inactive compounds correctly identified, while the sensitivity is determined by the proportion of active compounds correctly identified. The accuracy is defined as the ratio of correct predictions.

The CP models were evaluated at a significance level ε of 0.2, i.e., at a confidence level (1 – ε) of 0.80. The set of predicted classes at this confidence level will contain the true class label in at least 80% of the cases (for valid models). This significance level was selected because it usually offers an adequate trade-off between efficiency and validity.^57,58^

The difference in performance between models with distinct descriptors was evaluated with the nonparametric Mann–Whitney U test.^59^ For each pair of models compared, the distribution of values obtained in the different CV runs for a given performance metric (e.g., efficiency) was given as input in the “mannwhitneyu” function implemented in SciPy.^60^

## Results and Discussion

In this study, we investigated if, and to what extent, the consideration of predicted bioactivities can improve the performance of in silico models for the prediction of the in vivo toxicity endpoints MNT, DILI, and DICC. To this end, we first trained CP models for 373 biological assays and applied them on the in vivo endpoint data sets for deriving the predicted bioactivities. For training the models for the three in vivo endpoints, we embedded three types of RF models in CP frameworks: (a) CHEM models based exclusively on chemical descriptors, (b) BIO models based exclusively on (predicted) bioactivity descriptors, and (c) CHEMBIO models based on the combination of both types of descriptors.

### Chemical Space Analysis

In order to develop an understanding of the chemical space represented by the training data from the three in vivo endpoints (MNT, DILI, and DICC), we compared the overlap of the chemical space between the in vivo endpoint data sets and three reference data sets. The overlap between data sets serves as an indication of the relevance of models trained on the in vivo data sets for different chemical domains (pesticides, cosmetics, and drugs). The reference data sets represent pesticides (2417 compounds from the EPA’s Office of Pesticide Programs), cosmetics (4503 cosmetics ingredients from the COSMOS database), and drugs (10,087 approved, experimental, or withdrawn drugs from DrugBank).

We found that the MNT data set covers 16% of the pesticides reference set, 10% of the cosmetics reference set, and 8% of the drugs reference set, considering exact matches only (exact matches defined as any pair of compounds with a Tanimoto coefficient of 1.00; Table 3). The DICC data set covers 34% of the drugs reference set but just 7 and 6% of the cosmetics and pesticides reference sets, respectively. The lowest coverage rates were observed for the DILI data set (as it is also the smallest data set), with just 6, 2, and 1% for the drugs, pesticides, and cosmetics reference sets, respectively.

**Table 3:** Percentage of Compounds in the Reference Data Sets Covered by Compounds in the Three In Vivo Endpoint Data Sets (MNT, DILI, DICC) at Given Similarity Thresholds

		endpoint		
parameter	Tanimoto coefficient threshold^a^	MNT	DILI	DICC
% coverage pesticides	1.0	16	2	6
	≥0.8	17	2	7
	≥0.6	29	3	11
	≥0.4	62	10	36
	≥0.2	99	85	97
% coverage cosmetics	1.0	10	1	7
	≥0.8	14	1	9
	≥0.6	29	3	17
	≥0.4	68	17	58
	≥0.2	99	89	99
% coverage drugs	1.0	8	7	34
	≥0.8	9	8	37
	≥0.6	16	15	51
	≥0.4	40	34	73
	≥0.2	99	96	100

a Tanimoto coefficients calculated from binary Morgan fingerprints (1024 bits and radius 2).

For assessing the structural relationships between the active and inactive compounds present in the MNT, DILI, and DICC in vivo data, we referred to PCA. The PCA was performed on selected interpretable molecular descriptors, which describe, e.g., the number of bonds, rings, and particular types of atoms in a molecule, or the polarity and solubility of the compounds (Table S4). The three in vivo toxicity data sets were combined (containing 4987 compounds) and used to perform the PCA.

**Figure 3:**
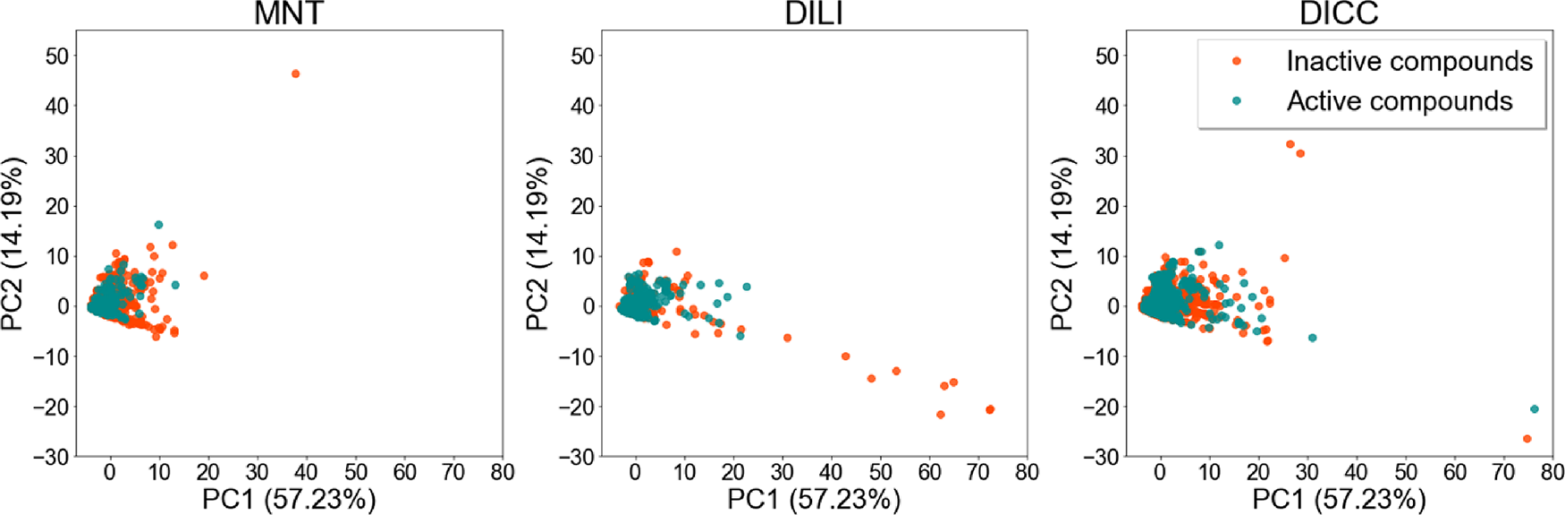
Principal component analysis based on a selection of interpretable molecular descriptors generated with RDKit on the merged in vivo toxicity data sets. Inactive compounds are colored in red and active compounds in green. The variance explained by the first two principal components is indicated in the axes.

The PCA plots reported in Figure 3 indicate that the physicochemical properties of the active and inactive compounds of the individual in vivo endpoint data sets are mostly similar, with only a few outliers. Outliers with high values for the first principal component (PC1, *x* axis) are molecules with high molecular weight. Outliers with low values in the second component of the PCA (PC2, *y* axis) are mostly acyclic and polar, while molecules with high values on this axis have a high number of rings. Most outliers are inactive on the three investigated endpoints. The loadings plots (indicating how strongly each descriptor influences a principal component) are provided in Figure S1.

In order to investigate the chemical space with regard to the full set of descriptors used for model training, we utilized UMAP to compare the two-dimensional projections of the CHEM and CHEMBIO descriptor sets. UMAP conducts a dimension reduction of the data while maintaining the pairwise distance structure among all samples. In general, no clear separation of activity classes emerged for any of the three endpoints. Moreover, no significant difference was observed in the projections derived from the two descriptor sets regarding their ability to cluster compounds with different activity labels. The resulting UMAP plots are provided in Figure S2.

**Figure 4:**
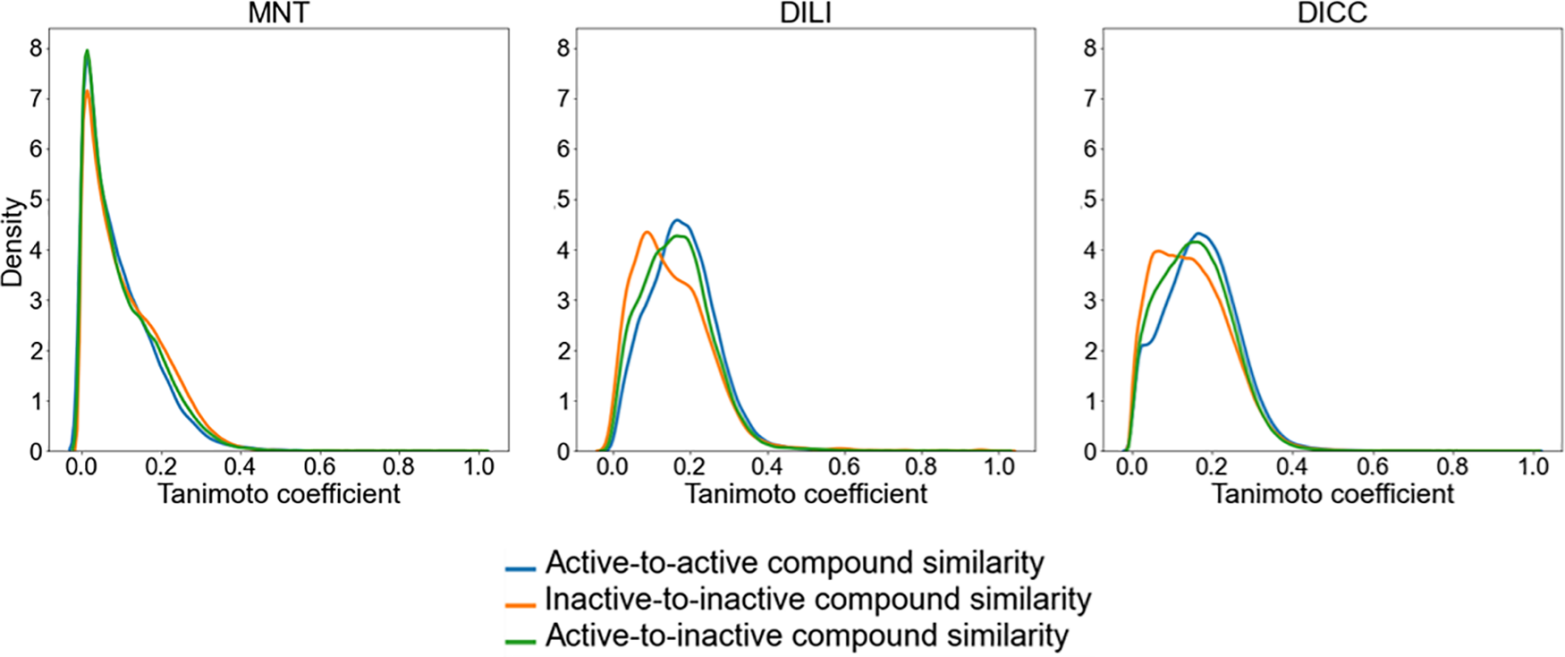
Distribution of pairwise Tanimoto coefficients based on atom-pair fingerprints for three types of compound pairs: (a) active-to-active (blue), (b) inactive-to-inactive (orange), and (c) active-to-inactive (green).

The structural diversity within the individual compound sets was determined based on the distribution of pairwise Tanimoto coefficients (based on atom-pair fingerprints)^61^ among (a) all pairs of active compounds, (b) all pairs of inactive compounds, and (c) all pairs consisting of one active and one inactive compound (Figure 4). For the three in vivo endpoints, the distribution of pairwise compound similarities shows a tailing toward low similarities for the three sets of compounds (a, b, and c), indicating a high molecular diversity in the data sets. It is also shown that compounds in one class are not more similar to each other than they are to compounds of the other class, since the distribution of similarities of the three subsets is in all cases comparable.

Hence, the classification of compounds in the active and inactive classes based only on their structural similarity is not straightforward and complementary information may be necessary for in silico methods to be able to differentiate between classes.

### Performance of CP Models for Deriving the Predicted Bioactivities

With the aim to improve the predictive performance for in vivo toxicity endpoints, we included information about the outcome of the compounds in biological assays (obtained from the ToxCast database, eMolTox, eChemPortal, and other publications) as input for the in vivo toxicity CP models. To avoid increased sparsity of the data due to missing experimental values, a fingerprint based on predicted bioactivities was developed. More specifically, for each of the 373 collected biological assay data sets, a bioactivity CP model was trained on molecular fingerprints and physicochemical property descriptors (see Materials and Methods for details).

CP models are a type of confidence predictor that use the predictions made by the model on a set of compounds with known activities (calibration set) to rank and estimate the certainty of the predictions for new compounds^57^ (see Materials and Methods section for details). These models output a set of labels (instead of only one label), which can contain one class (active or inactive), both classes, or none of them. Therefore, two important metrics for the evaluation of CP models are the validity, which measures the ratio of prediction sets containing the correct label (i.e., the “both” class is always correct), and the efficiency, which measures the ratio of single class predictions. Furthermore, the quality of the single class predictions (covered by the AD of the model) can be evaluated with common metrics like the F1 score or the MCC. The performance of models developed in this work was evaluated on the validity, efficiency, and F1 score results referring to mean values obtained by 5-fold CV at a significance level ε of 0.2 (Table S6). The MCC, specificity, sensitivity, and overall and class-wise mean accuracies of the single class predictions are also provided in Table S6.

The AD of ML models defines the region in chemical space where the model makes predictions with a given reliability. Depending on the focus of the study, there are different ways to define the AD. For example, unusual compounds or unreliable predictions can be flagged, assuming that they are likely outside the aforementioned region. In our case, error rate reduction is the focus of defining an AD; hence, it is mandatory to use confidence measures to identify objects close to the decision boundary and reject their predictions. A large benchmark study from Klingspohn et al. concluded that built-in class probability estimates performed constantly better than the alternatives (e.g., distance measures) in terms of error reduction.^62,63^ In the current study, we are using the RF prediction score (best confidence measure for RF) as nonconformity measure for the CP. Hence, it is expected that no other nonconformity measure (or method) will outperform the prediction score to estimate the confidence of the predictions.

**Figure 5:**
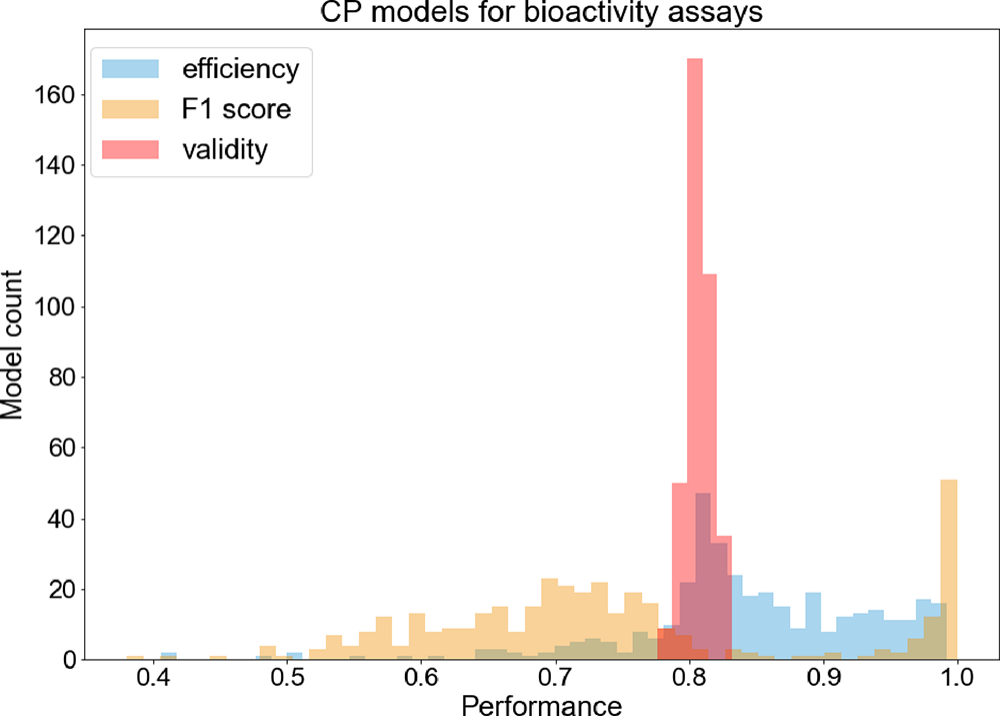
Histogram of the performance distribution of the CP models for the biological assays. All models were valid but their efficiencies and F1 scores showed a high degree of variability.

**Figure 6:**
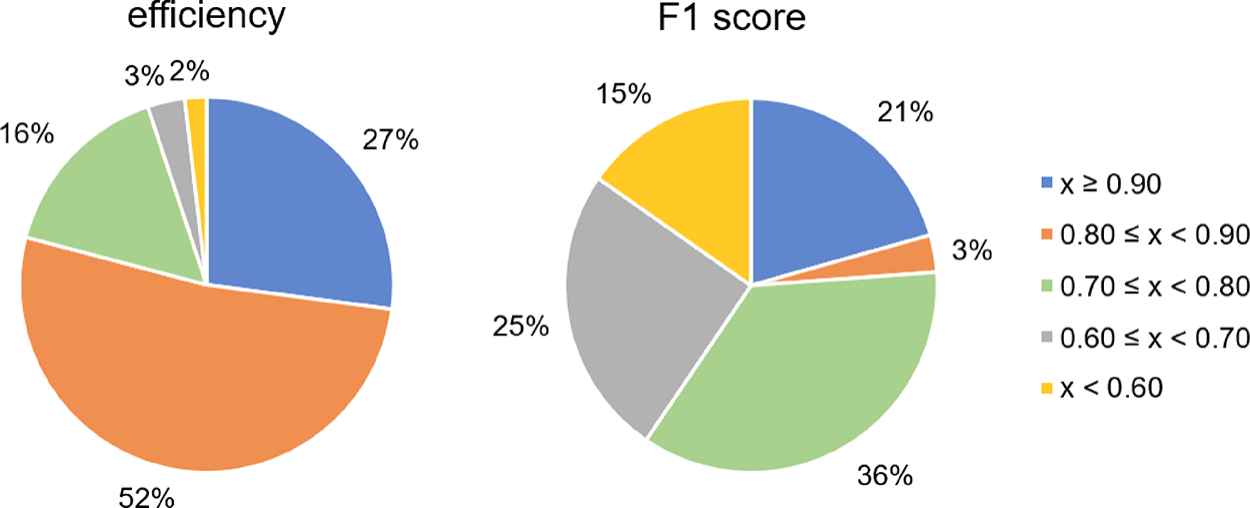
Percentage of the 373 bioactivity CP models showing mean efficiencies and mean F1 scores in the four given ranges.

All 373 bioactivity CP models showed adequate mean validities for the given significance level (for which the expected validity is 0.80) that ranged from 0.78 to 0.83 (Figure 5) and thus obtained the defined error rate. The mean efficiency values and F1 scores spread over a wider range. There were 19 CP models (5%) with mean efficiencies lower than 0.70 (Figure 6). The lowest mean efficiency (0.41) was obtained for the ToxCast assay “ATG Ahr CIS dn”. On the other hand, mean efficiencies higher than 0.90 were achieved for 101 CP models (27%), where the highest mean efficiency of 0.99 was obtained for the two eMolTox assays “Substrates of cytochrome P450 2C19” and “Differential cytotoxicity (isogenic chicken DT40 cell lines)”, and the two ToxCast assays “TOX21 ERa LUC VM7 antagonist 0.1nM E2” and “TOX21 SBE BLA antagonist ratio”. Hence, the ratio of single class predictions obtained by the bioactivity CP models was relatively high and only in a few cases the models showed poor efficiencies. In general, the models with the lowest mean efficiency had highly imbalanced classes and a low number of active compounds, while the contrary was observed for the models showing the highest mean efficiencies.

Seventy-seven models (21%) obtained F1 scores higher than 0.90, indicating a very good performance of these models on the single class predictions. There were 149 CP models (40%) with mean F1 scores lower than 0.70. Only for 15% of all models, the mean F1 scores were lower than 0.60, indicating poor performance. The worst-performing model was that for the ToxCast assay “ATG Ahr CIS dn” (mean F1 score of 0.38) and the best-performing ones for the eMolTox assays “Modulator of Neuropeptide Y receptor type 1”, “Modulator of Urotensin II receptor”, and “Agonist of Liver X receptor alpha” (F1 score of 1.00). One explanation for the good predictivity could be the fact that the chemical space of the active and inactive compounds is well differentiated (PCA plots of the chemical space of these data sets are shown in Figure S3). The classification of these compounds might therefore be easier than for data sets with more similar compounds between classes.

The performance of all CP models for the biological assays can be found in the Supplementary Information (Table S6).

### In Vivo Toxicity CP Model Performance

The in vivo toxicity CP models were trained on three sets of descriptors: (i) the chemical descriptor set (“CHEM”) comprising physicochemical features and the molecular fingerprint; (ii) the bioactivity descriptor set (“BIO”) containing the predicted *p*-values for the biological endpoints; and (iii) the “CHEMBIO” descriptor set, which contains all features from both the CHEM and the BIO descriptor sets.

The number of features in the CHEM descriptor set (2171 features) is almost three times higher than the number of features of the BIO descriptor set (746 features), and together, they add up to 2917 features. The underrepresentation of bioactivity features in the CHEMBIO descriptor set and, more generally, the high number of total features could lead to a dilution of relevant information in the high-dimensional feature space. Moreover, since no prefiltering has been applied to the BIO descriptor set, some features may be redundant or less relevant for the specific in vivo endpoints. In order to test whether a reduction of the feature space could increase the performance of the in vivo toxicity CP models, we introduced a feature selection procedure based on a lasso model (which assigns coefficients, i.e., weights, to all features) that we applied prior to model training (see Materials and Methods for details).

With each of the CHEM, BIO, and CHEMBIO descriptor sets, two types of models were trained: (i) baseline models based on all features of the respective descriptor set (only filtering out those features with low variance; see Materials and Methods for details) and (ii) models based on a subset of features selected with a lasso model (built on the feature subset after the variance filter). For the model training, only those features with coefficients higher than zero in the lasso model were selected (see Materials and Methods for details).

The models based on the preselected set of features (based on (ii) lasso procedure) generally performed better (details will be discussed together with the individual in vivo endpoint performances below) and also present the computational advantage that only the *p*-values for the selected biological assays need to be computed to build the bioactivity descriptor for new compounds. Therefore, in the following paragraphs, only the results of these models will be further discussed. The results from the baseline models without feature selection with lasso (as described in (i)) are presented in Figure S3 and Table S7. All models were evaluated on the mean validity, efficiency, and F1 score (on the single class predictions) over 5-fold CV at a significance level ε of 0.2. The MCC is presented in Table 4 (see discussion in the next paragraph); specificity, sensitivity, and overall and per class accuracy data are provided in Table S8. The differences in the performance among models with different descriptors are evaluated with a Mann–Whitney U test at a *p*-value <0.05.

**Table 4:** Average Performance of the CP Models Generated from a Selected Set of Features^a^

endpoint	descriptor	validity	STD validity	efficiency	STD efficiency	F1 score	STD F1 score	MCC	STD MCC
MNT	CHEM	0.77	0.02	0.76	0.05	0.61	0.02	0.28	0.05
	BIO	**0.82**	0.03	0.81	0.05	**0.70**	0.03	**0.46**	0.06
	CHEMBIO	0.81	0.03	**0.85**	0.03	**0.70**	0.03	0.44	0.07
DILI	CHEM	0.78	0.05	**0.91**	0.04	0.74	0.05	0.49	0.09
	BIO	**0.81**	0.04	0.83	0.07	0.76	0.04	0.53	0.07
	CHEMBIO	**0.81**	0.03	0.88	0.04	**0.77**	0.03	**0.55**	0.06
DICC	CHEM	0.79	0.02	0.84	0.02	0.72	0.03	0.46	0.05
	BIO	0.79	0.02	**0.96**	0.02	0.81	0.01	0.63	0.02
	CHEMBIO	0.79	0.02	0.94	0.01	**0.82**	0.01	**0.65**	0.03

a Mean and standard deviation (STD) calculated over a 5-fold CV. The highest mean per metric and endpoint is highlighted (bold).

It is important to consider the inherent noise and errors in experimental data, which sets the upper limit for the models’ performance, as a model can only be as good as the data it is trained on.^64^ Hence, models trained on chemical descriptors only, which already achieve high performance rates, may not benefit from the addition of bioactivity fingerprints, as the noise in the data may be the bottleneck in these cases. Unfortunately, there is no information available on the noise in the data sets under investigation. Since studies such as that by Zhao et al.^65^ have shown that low levels of noise are often tolerated by models while the removal of suspicious data points often decreases model performances and causes overfitting issues, we decided to not attempt to identify and remove noise in the data.

To evaluate the influence of the predicted bioactivities on model performance, the results of the in vivo toxicity CP models (including feature selection with lasso) based on the CHEM, BIO, and CHEMBIO descriptor sets were analyzed for each of the three in vivo endpoints.

**Figure 7:**
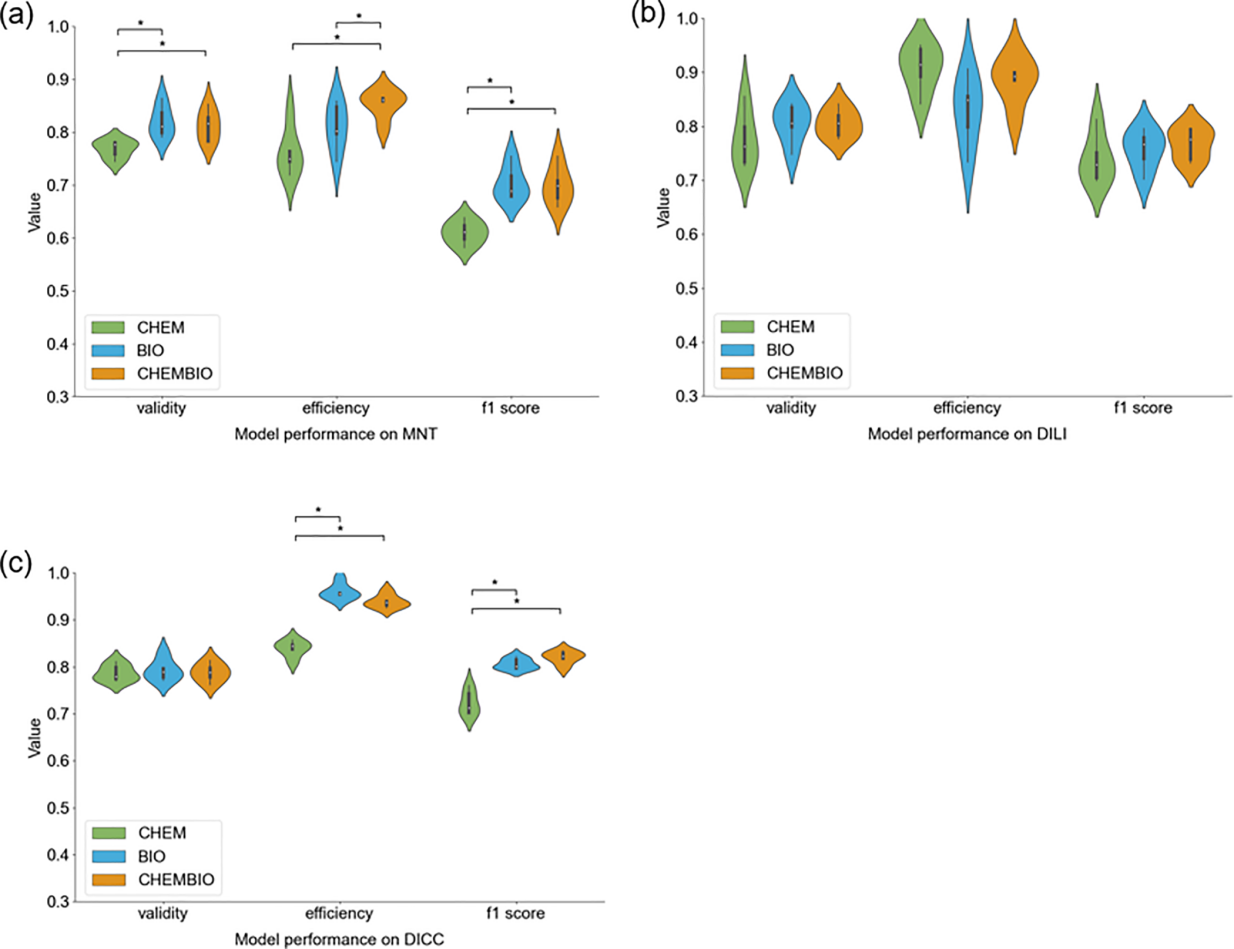
Distribution of the validity, efficiency, and F1 score values obtained within the 5-fold CV framework for the (a) MNT, (b) DILI, and (c) DICC CP models built on the different descriptor sets after feature selection. The CHEM descriptor set includes the molecular fingerprint and physicochemical descriptors; the BIO descriptor set includes the predicted *p*-values for a set of biological endpoints (bioactivity descriptor); the CHEMBIO descriptor set includes the previous two descriptor sets. Significant differences in the distribution (*p*-value <0.05) are denoted by a star.

For the MNT endpoint, the mean validities obtained by the two models including the BIO descriptor set (0.82 (±0.03) with the BIO and 0.81 (±0.03) with the CHEMBIO descriptor sets) were significantly higher than the validity of the model trained on the CHEM descriptor set alone (mean validity of 0.77 (±0.02); Figure 7, Table 4). While the validity of the model based on the CHEM descriptor set (0.77 ± 0.02) was lower than the expected validity at a significance level of 0.2 (i.e., expected validity of 0.80), the validity could be restored by adding the bioactivity descriptors (in the BIO and CHEMBIO descriptor sets). The mean efficiency obtained with the CHEMBIO descriptor set (0.85 ± 0.03) was significantly higher than the one obtained with the CHEM descriptor set alone (0.76 ± 0.05) but also higher than with the BIO descriptor set (0.81 ± 0.05) only. The two models including the BIO descriptor set significantly increased the predictive performance of the single class predictions, as reflected by the F1 score. More specifically, the model based on the CHEM descriptor set yielded a mean F1 score of 0.61 (±0.02), while the models based on the BIO and CHEMBIO descriptor sets both obtained a mean F1 score of 0.70 (±0.03). Thus, the model based on the CHEMBIO descriptor set not only increased the number of single class predictions but also the accuracy of these predictions.

The analysis of the number and type of the features selected with lasso for the models based on the CHEMBIO descriptor set showed that a total of 157 features were selected, 30 of which were bioactivity features (19%). Of the 15 features with the highest lasso coefficients, seven were bioactivity features and eight are chemical features (Table S10). Compared to the models without feature selection, the efficiency of the CHEMBIO MNT model including feature selection was significantly higher (0.07 higher mean efficiency). Otherwise, the difference in the performance between models with and without feature selection (only comparing models with the same descriptor set) was not significant.

The DILI models obtained mean validities between 0.78 (±0.05; with the CHEM descriptor set) and 0.81 (±0.04 with the BIO and ±0.03 with the CHEMBIO descriptor sets). The distribution of efficiencies within the CV from models trained on the different descriptor sets was not significantly different. However, the mean efficiencies ranged from 0.83 (±0.07; with the BIO descriptor set) to 0.91 (±0.04; with the CHEM descriptor set; Figure 7). The mean F1 score based on the single class predictions was also comparable among the three models and was between 0.74 (±0.05) with the CHEM descriptor set and 0.77 (±0.03) with the CHEMBIO descriptor set. Although there is no model for DILI that outperforms the others, the models including biological features (CHEMBIO and BIO) have a slightly higher mean validity and F1 score (while a lower number of single class predictions is obtained compared to the model trained on the CHEM descriptor set). Thus, both the BIO and CHEM descriptor sets may contain relevant—but not complementing—information for the prediction of the DILI endpoint. In the model based on the CHEMBIO descriptor set, 648 features were selected by the lasso model, 59 of which were bioactivity features (9%). The smaller percentage of bioactivity features (compared to the number of features in the MNT model) among the selected features also reflects the fact that including the bioactivity descriptor set did not improve the performance of the models significantly for this endpoint. Nevertheless, among the 15 features with the highest lasso coefficients, nine were bioactivity features and six were chemical features (Table S10). Compared to the models without feature selection by lasso, the efficiencies of the BIO and CHEMBIO models were significantly increased (up to 0.08 higher mean efficiency).

In the case of the DICC endpoint, the models based on each of the three different descriptor sets yielded mean validities of 0.79 (±0.02). The models trained on the BIO and CHEMBIO descriptor sets showed significantly higher efficiencies (0.96 ± 0.02 and 0.94 ± 0.01, respectively) than the model trained on the CHEM descriptor set (0.84 ± 0.02, Figure 7). Not only the ratio of single class predictions (i.e., efficiency) was improved in the models including the BIO descriptor set but also the quality of these predictions. The two models including the BIO descriptor set obtained significantly higher F1 scores (mean F1 score of 0.81 (±0.01) with the BIO and 0.82 (±0.01) with the CHEMBIO descriptor sets) than the model based on the CHEM descriptor set (mean F1 score of 0.72 (±0.03)). The significantly better performance of the DICC models making use of the BIO descriptor set over the DICC models based solely on CHEM descriptors is also reflected in the nature of the features selected by lasso from the CHEMBIO descriptor set: among the 666 features selected, 101 are bioactivity features (15%). Furthermore, the bioactivity features were assigned high coefficients by the lasso model, and from the top 50 features (ranked after the mean coefficient), 34 belong to the bioactivity descriptor set (15 out of the top 15 features are bioactivity features; Table S10). Compared to the models without feature selection, the efficiencies of the two models including the BIO descriptor set decreased when the feature selection was included (up to 0.03 lower mean efficiency). Also, the mean F1 score of the model trained on the CHEM descriptor set decreased by 0.04 when including the feature selection procedure. One possible explanation for the decrease in performance is the potential overfitting of the models without feature selection to the training data due to the high number of features.

In summary, it was shown that the addition of bioactivity descriptors in the form of predicted *p*-values for a set of biological assay outcomes can improve the predictive ability of CP models with regard to the number of single class predictions as well as to the quality of these predictions. However, this effect and its magnitude were endpoint-dependent and not achieved in all cases. It was also shown that including feature selection before training, the models can help to discard irrelevant features favoring those more relevant for the specific endpoint.

### Comparison with Existing Models

Several in silico models for MNT, DILI, and DICC are described in the literature (Table 5). However, to our knowledge, no CP models have been previously developed for these endpoints. Note that the studies cannot be directly compared given differences in underlying data and techniques. Also, the evaluation of the models differs since the quality of the predictions of CP models is in general evaluated on single class predictions only. However, considering existing models can help to put the results of this study into context.

**Table 5:** Summary of Model Performances of the ChemBioSim Models and Existing Methods

endpoint	model	mean sensitivity	mean specificity	evaluation	modeling approach	comments
MNT	Yoo et al.	0.54–0.74	0.77–0.93	5% leave-many-out	Leadscope Enterprise and CASE Ultra software	variations related to different modeling approaches
	our method	0.78	0.76	5-fold CV	CP built on RF models	CHEMBIO model with feature selection
DILI	Ancuceanu et al.	0.83	0.66	nested CV	meta-model with a naïve Bayes model trained on output probabilities of 50 ML models	
	our method	0.78	0.78	5-fold CV	CP built on RF models	CHEMBIO model with feature selection
DICC	Cai et al.	0.69–0.75	0.72–0.81	5-fold CV	combined classifier using neural networks based on four single classifiers	results refer to five cardiological complications endpoints evaluated independently
	our method	0.83	0.86	5-fold CV	CP built on RF models	CHEMBIO model with feature selection

Yoo et al.^33^ recently collected data sets for MNT in mice and rats, containing 1001 and 127 compounds, respectively. They developed statistical-based models with the Leadscope and CASE Ultra software combined with different balancing techniques for the mouse data set based on chemical features and structural alerts (functional groups or substructures frequently found in molecules eliciting a determined biological effect). Their best model with regard to specificity (i.e., the proportion of inactive compounds correctly identified) on a 5% leave-many-out framework yielded a mean specificity of 0.93 but a mean sensitivity (i.e., the proportion of active compounds correctly identified) of only 0.54. The model with the highest sensitivity (and also with the most balanced sensitivity-to-specificity ratio) obtained a mean specificity of 0.77 and a mean sensitivity of 0.74. To train our MNT CP models, we combined the mouse and rat data sets from Yoo et al. and added further data sources (see Materials and Methods section) to obtain a data set with 1791 compounds. For comparison, the specificity and sensitivity values obtained by our models trained on the CHEMBIO descriptor set including feature selection with lasso were also calculated (Table 5). The CHEMBIO model for the MNT endpoint yielded a mean specificity of 0.76 and a mean sensitivity of 0.78. Thus, compared to the most balanced model of Yoo et al., our model showed a slightly higher sensitivity and comparable specificity on a significantly larger data set (790 additional compounds).

Several in silico models with adequate predictive performance have already been reported for the DILI endpoint.^66−68^ In a recent study based on the same data set as our models, Ancuceanu et al.^68^ built 267 different models combining feature selection techniques with ML algorithms. Meta-models using the output of 50 ML models as input for a final model were developed. Their meta-model with the highest balanced accuracy (0.75) evaluated in a nested CV was built training a naïve Bayes model on output probabilities of 50 ML models. This model yielded a mean specificity of 0.66 and a mean sensitivity of 0.83. In comparison, our CHEMBIO DILI model yielded a much more balanced sensitivity-to-specificity ratio. The mean specificity and sensitivity obtained by our model were both 0.78.

Although in silico models for cardiological complications are more scarce, Cai et al.^35^ compiled data sets for five different cardiological complications (hypertension, arrhythmia, heart block, cardiac failure, and myocardial infarction), on which our DICC data set is based, and developed a combined classifier for each of the five endpoints. These classifiers yielded mean specificities between 0.72 and 0.81 and sensitivities between 0.69 and 0.75 (depending on the endpoint). Our CHEMBIO model for the DICC endpoint yielded a mean specificity of 0.86 and a mean sensitivity of 0.83, thus increasing the performance observed for the previous models (especially with regard to the sensitivity).

Overall, our models yielded a high balanced sensitivity-to-specificity ratio and often generally good performance. It should be considered that the existing models used for comparison were built on complicated and highly optimized model architectures for the studied endpoint, while in this study, we used simple RF models without hyperparameter optimization embedded in a CP framework for the predictions with the aim of comparing the different descriptors.

### Analysis of Feature Importance to Discover Biological Relationships

Understanding which bioactivity features are most important for the prediction can help to identify the most relevant assays for an endpoint and to discover unknown biological relationships. From the complete CHEMBIO descriptor set (i.e., the descriptor set without feature selection with lasso), we analyzed the 15 descriptors that were assigned the highest feature importance values by the RF model. The reason for using the complete set of CHEMBIO descriptors instead of the subset of features selected by the lasso method (which generally yields better performing models) is that the lasso model discards highly correlated features during the feature selection. Therefore, feature importance analysis involving a descriptor preselection with lasso may lead to an underestimation of the importance of some of the features.

The RF model for the MNT endpoint ranked the features from (i) the AMES assay, (ii) the eMolTox assay for mutagenicity, and (iii) the eMolTox assay for agonism on the p53 signaling pathway as the most important features (Table S9). These three in vitro assays are known to be biologically related to the MNT endpoint: the AMES and mutagenicity assays evaluate the genotoxic potential of compounds in vitro by measuring the capability of substances to induce mutations in bacterial strains. DNA damage leading to these gene mutations could also cause the chromosome aberrations observed in the MNT.^69^ The tumor suppressor p53 has the capacity of preventing the proliferation of cells with a damaged genome and is also referred to as “the Guardian of the Genome”.^70^ The p53 signaling pathway is activated i.a. when DNA damage accumulates in a cell. As a result, a mechanism of cell cycle arrest, cellular senescence or apoptosis is initiated. Since genotoxic damage is one of the primary triggers of the activation of the p53 signaling pathway, the detection of agonism of the p53 pathway could be an indication of the genotoxic activity of a compound, which could also lead to micronuclei formation in vivo.^71^ The contribution of the p53 signaling pathway for the prediction of MNT in vivo is highlighted by the high feature importance assigned to features corresponding to further assays related to this endpoint (ToxCast assays “TOX21 p53 BLA p3 ratio,” “TOX21 p53 BLA p5 ratio,” and “TOX21 p53 BLA p2 ratio” (each measuring the ratio of two measurements with the inducible beta lactamase (BLA) reporter); Table S9). Also the biological function of the constitutive androstane receptor (CAR) and aryl hydrocarbon receptor (AhR) could explain the high importance assigned by the model to the ToxCast assay “TOX21 CAR antagonist” and the eMolTox assay “Activator the aryl hydrocarbon receptor (AhR) signaling pathway.” The AhR and the CAR are ligand-activated transcription factors functioning as sensors of xenobiotic compounds. Upon activation of these receptors, i.a. the expression of enzymes involved in the metabolism of xenobiotic compounds, is upregulated.^72,73^ The downregulation of enzymes detoxifying compounds (or their metabolites) mediated by CAR antagonists, as well as the AhR-mediated upregulation of enzymes activating compounds to form genotoxic metabolites seem to contribute to the observed effects in the MNT. The remaining features among the 15 most important features for MNT are related to the eMolTox assay “Antagonist of the farnesoid-X-receptor (FXR) signaling pathway.” The FXR, also called bile acid receptor, is a nuclear receptor that regulates, among other things, bile acid and hepatic triglyceride levels.^74^ Its possible biological relationship with genotoxicity has not been reported so far (to the best of our knowledge). Comparing the features with the highest feature importance values with RF to the features with the highest lasso coefficients during feature selection (Table S9 and Table S10), an overlap of the assays for AMES, the p53 signaling pathway, and the CAR antagonism was observed, highlighting the relevance of these biological endpoints for the prediction of MNT.

Although in the case of DILI the performance of the RF models making use of bioactivity descriptors was not superior (see Table 4) over that of the models trained on chemical descriptors only, 14 out of the 15 top-ranked features were bioactivity features. The highest feature importance was obtained for a chemical descriptor (smr VSA10) that captures polarizability properties of compounds. The bioactivity features ranked at positions 3 and 4 are the two p-values (of the active and inactive classes) for human oral bioavailability, respectively. Since any compound must be absorbed and distributed in order to be able to elicit any kind of biological response, bioavailability is essential to induce liver injury. Moreover, orally administered substances undergo a hepatic first pass before they become systemically available. Other than that, several features related to modulators of G protein-coupled receptors were of high importance (see Table S9). Despite the lack of a clear biological relationship between liver injury and opioid receptors (kappa, mu and delta) or muscarinic acetylcholine receptors (M2, M3, M4 and M5), the activity of compounds against these receptors showed high predictivity for DILI. Between the features with the highest feature importance values for RF and the features with the highest lasso coefficients (Table S10) we found an overlap of descriptors for the bioavailability, mu opioid receptor, and muscarinic acetylcholine receptor assays.

Consistent with the DILI model, also the DICC model assigned high ranks (rank 1 and rank 4) to the two features related to human oral bioavailability (i.e., *p*-values for the active and inactive classes). The importance of these features is plausible, as substances first need to be absorbed in order to be able to elicit any response. We also found the ToxCast assay “TOX21 ERa LUC VM7 agonist”, an assay for detecting agonists of the estrogen receptor alpha, to have a high relevance value assigned by the DICC RF model. There is evidence about the important correlation between estrogen levels and cardiovascular diseases.^75^ The cardioprotective effects shown by estrogen derive from the increase in angiogenesis and vasodilation as well as the decrease in oxidative stress and fibrosis. Another feature that was assigned a high importance is agonism on the retinoid X receptor (RXR; eMolTox assay “Agonist of the RXR signaling pathway” and ToxCast assay “TOX21 RXR BLA agonist”). Following its activation, RXR forms homo- or heterodimers with other nuclear receptors (e.g., thyroid hormone receptor), regulating the transcription of several genes and therefore playing a role in diverse body functions. It has been shown that the functionality of RXR influences, for example, the composition of the cardiac myosin heavy chain, thus affecting the correct functionality of the heart.^76^ The induction of phospholipidosis, a phospholipid storage disorder in the lysosomes, was also assigned a high importance value by the DICC RF model. There is still controversy whether phospholipidosis is a toxic or an adaptive response, as it does not necessarily result in target organ toxicity.^77^ However, a high percentage of compounds inducing phospholipidosis has been found to also inhibit the human ether-à-go-go-related gene (hERG),^78,79^ an ion channel that contributes to the electrical activity of the heart. Inhibitors of hERG can lead to fatal irregularities in the heartbeat (ventricular tachyarrhythmia).^80^ Another bioactivity that was of high importance for the prediction of cardiological complications is the agonism of the p53 signaling pathway (ToxCast assays “TOX21 p53 BLA p2 ratio” and “TOX21 p53 BLA p3 ratio”). As already mentioned, the p53 transcription factor is related to tumor suppressor mechanisms of the cell, but it also inhibits the hypoxia-inducible factor-1 (Hif-1) in the heart. Inhibition of Hif-1 hinders cardiac angiogenesis (i.e., the formation of new blood vessels). This hindrance presents a problem in cases of cardiac hypertrophy (an adaptive response to increased cardiac workload), as blood pressure overload can lead to heart failure.^81,82^ Recently, heart failure has also been related to DNA damage. Higo et al.^83^ showed that single-stranded DNA damage is accumulated in cardiomyocytes of failing hearts and that mice lacking DNA repair mechanisms are more prone to heart failure. This relationship between DNA damage and heart failure could also explain the high relevance assigned by the DICC RF model to the three features related to genotoxicity in cells lacking DNA damage response pathways (from the eMolTox assay “Differential cytotoxicity against isogenic chicken DT40 cell lines with known DNA damage response pathways - Rad54Ku70 mutant cell line” and the ToxCast assay “TOX21 DT40 657”). The comparison of the most important features for RF with the features assigned the highest coefficients by lasso showed an overlap of the descriptors for the bioavailability and estrogen agonism assays. Furthermore, other assays related to genotoxicity (and correlated with the ones with a high feature importance shown in Table S9) were also assigned high coefficients.

Apart from biological relationships, there are other factors that may influence the importance values assigned to the respective bioactivity features. One should keep in mind that predicted *p*-values are used for the representation of biological properties, not measured bioactivity values. This means that feature importance values are likely affected by the performance and applicability of the individual models used for predicting the *p*-values. For example, bioactivity features based on biological assay data sets with a strong overlap with the in vivo endpoint data sets could be favored by a model, as the predicted *p* -values for structurally similar compounds are likely more accurate (as they were also used to train the bioactivity model itself).

**Figure 8:**
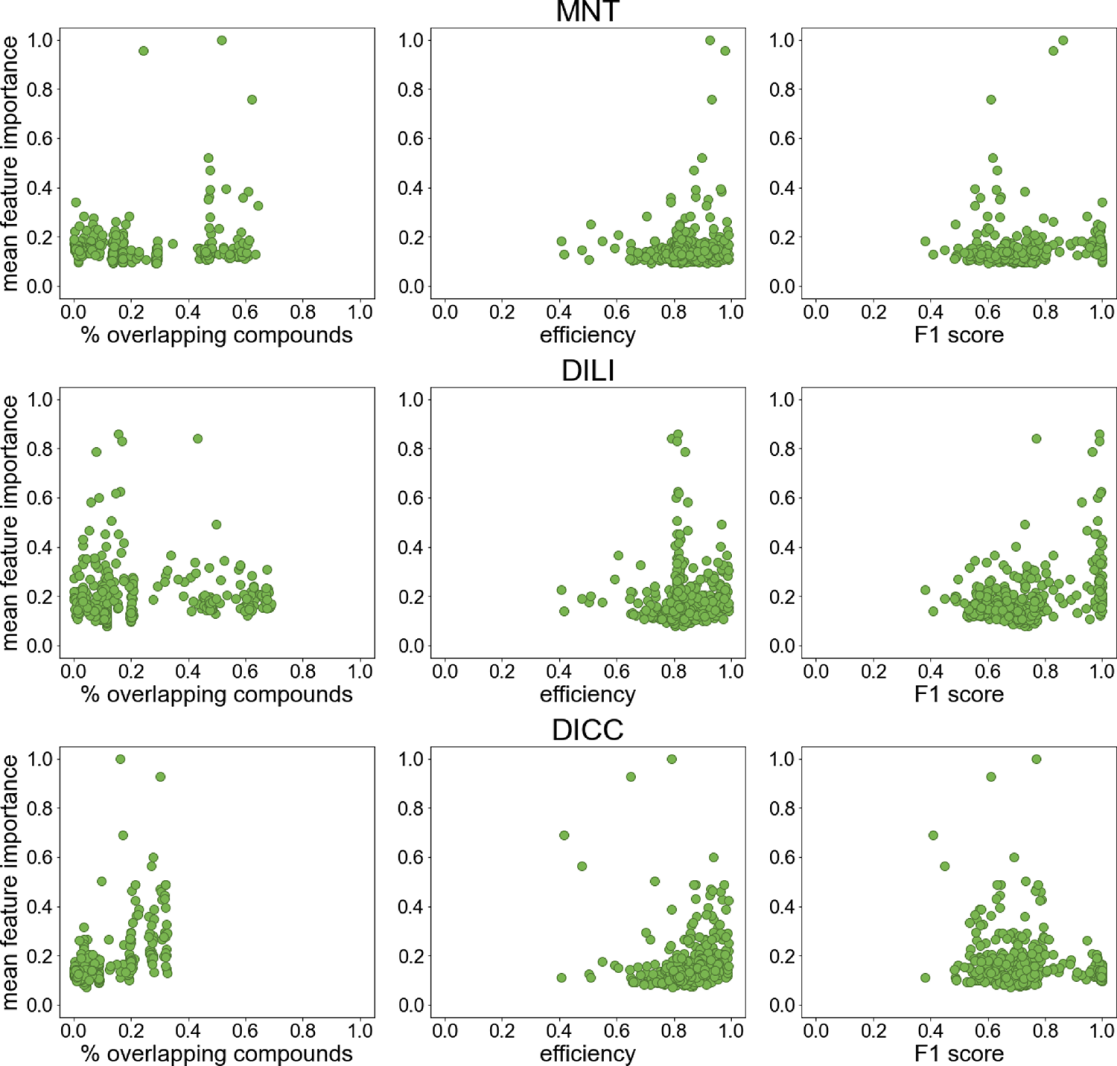
Mean feature importance reported by the RF model for the bioactivity descriptors in relationship with the percentage of overlapping compounds (of the in vivo data set), the efficiency and F1 score of the models for each biological assay. For each of the 373 biological assays, the highest mean feature importance of the two *p*-values used as descriptors (for the active and inactive classes of each assay) was taken. The feature importance values were normalized with a min-max normalization (from 0.01 to 1; see Materials and Methods section) for easier comparison.

Therefore, the overlap between the in vivo endpoint data set and the data sets of the selected biological assays, as well as the performance of the biological assay models, was analyzed to test possible correlations with the assigned feature coefficients. Overall, we observed no strong correlation between the extent of overlaps in the data and the assigned feature importance values. Also, no pronounced correlation between the performance of the bioactivity CP models and the feature importance values was observed (Figure 8), but bioactivity descriptors predicted with models showing lower efficiencies also often resulted in less important features.

The comparison between the data set overlap and model performance with the coefficients obtained during feature selection with the lasso model showed similar effects and correlations to the feature importance of the RF models discussed here (Figure S5).

In general, it was observed that the most predictive biological assays have a clear biological relationship with the corresponding in vivo endpoint. However, not all biological assays with a clear biological connection were assigned a high feature importance. Moreover, biological assays with a less obvious biological relationship were sometimes given a high relevance, as they may describe a more general behavior of the compounds in biological systems. These less obvious relationships could also reflect yet unknown effects and point to further lines of investigation.

## Conclusions

In this work, we have explored the potential of incorporating predicted bioactivities to improve the in silico prediction of in vivo endpoints beyond the level of accuracy reached by established molecular descriptors. More specifically, in the first part of this work, we collected 373 compound data sets with biological assay outcomes from the literature for modeling, and in the second part, we developed an elaborate conformal prediction framework in combination with the random forest algorithm, with the aim to identify the scope and limitations of the developed bioactivity descriptors for in vivo toxicity prediction on three selected in vivo endpoints (MNT, DILI, and DICC).

Overall, valid in vivo toxicity CP models could be produced with the different descriptors for all endpoints. For the MNT and DICC endpoints, the incorporation of predicted bioactivities was highly beneficial for the performance of the models. Compared to the models based only on chemical descriptors, the mean efficiencies of the models for MNT and DICC including bioactivity descriptors increased by 0.09 (from 0.76 to 0.85) and 0.12 (from 0.84 to 0.96), respectively. The mean F1 scores also increased by 0.09 (from 0.61 to 0.70) and 0.10 (from 0.72 to 0.82), respectively. The performance of the model for the DILI endpoint did not significantly improve by the integration of bioactivity descriptors, but a slight increase in the mean F1 score was also observed. The chemical and bioactivity descriptors may not complement each other for the prediction of DILI, which could explain the lower influence of the selected descriptor set on the performance. The prediction of the DILI endpoint may be especially challenging due to the nature of the data set, which has a reduced number of compounds and combines substances producing major and less severe effects in the active class. Further investigations are needed to determine how to improve the learning power of ML models for this endpoint.

In general, applying a feature selection procedure with a lasso model prior to model training with RF increased the mean efficiency of the models (up to 0.08 for the MNT and DILI endpoints). Feature selection proved especially beneficial in the models including the bioactivity descriptor set, as some biological assays may be redundant or not related to the in vivo endpoints.

The analysis of the most important features of the models based on the CHEMBIO descriptor set for each in vivo endpoint showed that generally these features had an explainable relationship with the biological mechanism eliciting the toxicity in vivo. For instance, some of the most important features for the MNT, an in vivo genotoxicity assay, are measuring genotoxicity in vitro or are involved in tumor suppressor mechanisms of the cells. In the case of the DILI and DICC endpoints, human oral bioavailability was ranked as one of the most important features, as bioavailability is an unavoidable requirement to elicit organ toxicity. Furthermore, the high feature importance assigned to assays with a less clear biological relationship could hint to unknown interactions that might help to better understand the toxic mechanisms.

The determination of which features will make the largest impact on the in vivo models prior to model development remains a difficult task since there are many factors influencing the relevance of the bioactivity features. However, using biological assays with known biological relevance for the in vivo endpoints is a well-suited approach. Also, for which in vivo endpoints the bioactivity descriptor will enhance the results cannot be predicted beforehand and may require evaluation case-by-case.

Overall, the approach presented in this work shows how the prediction of in vivo endpoints, which entail a high complexity due to all interactions taking place in biological systems, can be improved by the incorporation of bioactivity fingerprints. Moreover, the CP framework supporting the developed models also presents the advantage of intrinsically defining the applicability domain of these models and ensuring a defined error rate. Our approach also showed that bioactivity information can be included in the form of predicted probabilities, opening the possibility to apply these models directly on new compounds, without the need to fill their bioactivity profile experimentally. The bioactivity CP models for deriving the predicted bioactivities as well as the in vivo toxicity CP models trained on the different descriptor sets (and including feature selection with lasso) are freely available for download (https://doi.org/10.5281/zenodo.4761225).^84^

## Supporting Information Available

Loading plot of the PCA; UMAP projections for the three in vivo endpoints on the CHEM and the CHEMBIO descriptor sets; PCA of the biological assays with a mean F1 score of 1.0; distribution of the performance over 5-fold CV for the models for the three in vivo endpoints without feature preselection with lasso; scatter plots of lasso coefficients vs data set overlap and model performance of the models for the biological assays (PDF)
Download links, queries, and MD5 file checksum of the in vivo endpoint data sets; download links, queries, and MD5 file checksum of the biological assay data sets; data set information for the biological assays used to build the bioactivity descriptors; list of molecular descriptors used in principal component analysis; average performance of the CP models built on the biological assay data sets average performance of the CP for the three in vivo endpoints without feature preselection with lasso; top 15 features with the highest feature importance values for the three in vivo endpoints; top 15 features with the highest lasso coefficients for the three in vivo endpoints (ZIP)
KNIME workflow for the preparation of the molecular structures and calculation of the CHEM descriptors (ZIP)

## Notes

All data sets used in this study are publicly available and the original sources, as well as the processing workflow, are described in detail in the Materials and Methods section and Tables S1 and S2. Note that due to licensing issues of the original data, the data used in this study cannot explicitly be added as supporting information. The KNIME workflow used for preprocessing the structures and calculating the chemical descriptors is provided in the Supplementary Information. The workflow and parameters used for developing the models and necessary for reproducing the results are described in the Materials and Methods section. All bioactivity CP models (used for deriving the bioactivity descriptor) and in vivo toxicity CP models can be freely downloaded from https://doi.org/10.5281/zenodo.4761225. We believe that the scientific value of this study resides in researching the improvement of models for in vivo toxicity prediction with a fully automated workflow using predicted bioactivity fingerprints. Thus, this finding can be extrapolated to further endpoints and using other ML methods (and parameters).

## Notes

M.G.d.L., R.B., R.L., and M.M. are employed at BASF SE. U.N. performed research and served as a consultant for BASF SE.

## ABBREVIATIONS

AD: applicability domain
ADME: administration, distribution, metabolism, and excretion
AhR: aryl hydrocarbon receptor
BLA: beta lactamase
CA: chromosome aberration
CAR: constitutive androstane receptor
CCRIS: Chemical Carcinogenesis Information System
CP: conformal prediction
CTD: Comparative Toxicogenomics Database
CV: cross-validation
DICC: drug-induced cardiological complications
DILI: drug-induced liver injury
ECHA: European Chemicals Agency
EFSA: European Food Safety Authority
EPA: Environmental Protection Agency
FDA: U.S. Food and Drug Administration
FXR: farnesoid-X-receptor
hERG: human ether-à-go-go-related gene
Hif-1: hypoxia-inducible factor-1
ICH: International Council for Harmonisation of Technical Requirements for Pharmaceuticals for Human Use
MCC: Matthews correlation coefficient
ML: machine learning
MM: mammalian mutagenicity
MNT: micronucleus test
nc: nonconformity
NTP: National Toxicology Program
Papp: apparent permeability coefficient
PCA: principal component analysis
OECD: Organisation for Economic Co-operation and Development
RF: random forest
RXR: retinoid X receptor
STD: standard deviation
TSHR: thyroid stimulating hormone receptor
