# Supplementary material for: ChemBioSim: Enhancing Conformal Prediction of In Vivo Toxicity by Use of Predicted Bioactivities
Source: J Chem Inf Model. 2021 Jun 21;61(7):3255–72. doi: 10.1021/acs.jcim.1c00451 (PMC8317154; doi:10.1021/acs.jcim.1c00451)
Supplement: Supplementary file 1 — ci1c00451_si_001.pdf [file ci1c00451_si_001.pdf]

SUPPORTING INFORMATION

ChemBioSim: Enhancing Conformal Prediction of in vivo Toxicity by Use of Predicted Bioactivities

Marina Garcia de Lomana[1,2], Andrea Morger[3], Ulf Norinder[4], Roland Buesen[1], Robert Landsiedel[1], Andrea Volkamer[3], Johannes Kirchmair[2] and Miriam Mathea[1]**

[1] BASF SE, 67063 Ludwigshafen am Rhein, Germany

[2] Department of Pharmaceutical Sciences, Faculty of Life Sciences, University of Vienna, 1090 Vienna, Austria

[3] In Silico Toxicology and Structural Bioinformatics, Institute of Physiology, Charité Universitätsmedizin Berlin, Charitéplatz 1, 10117 Berlin, Germany

[4] MTM Research Centre, School of Science and Technology, Örebro University, SE-70182 Örebro, Sweden

* johannes.kirchmair@univie.ac.at;

miriam.mathea@basf.com

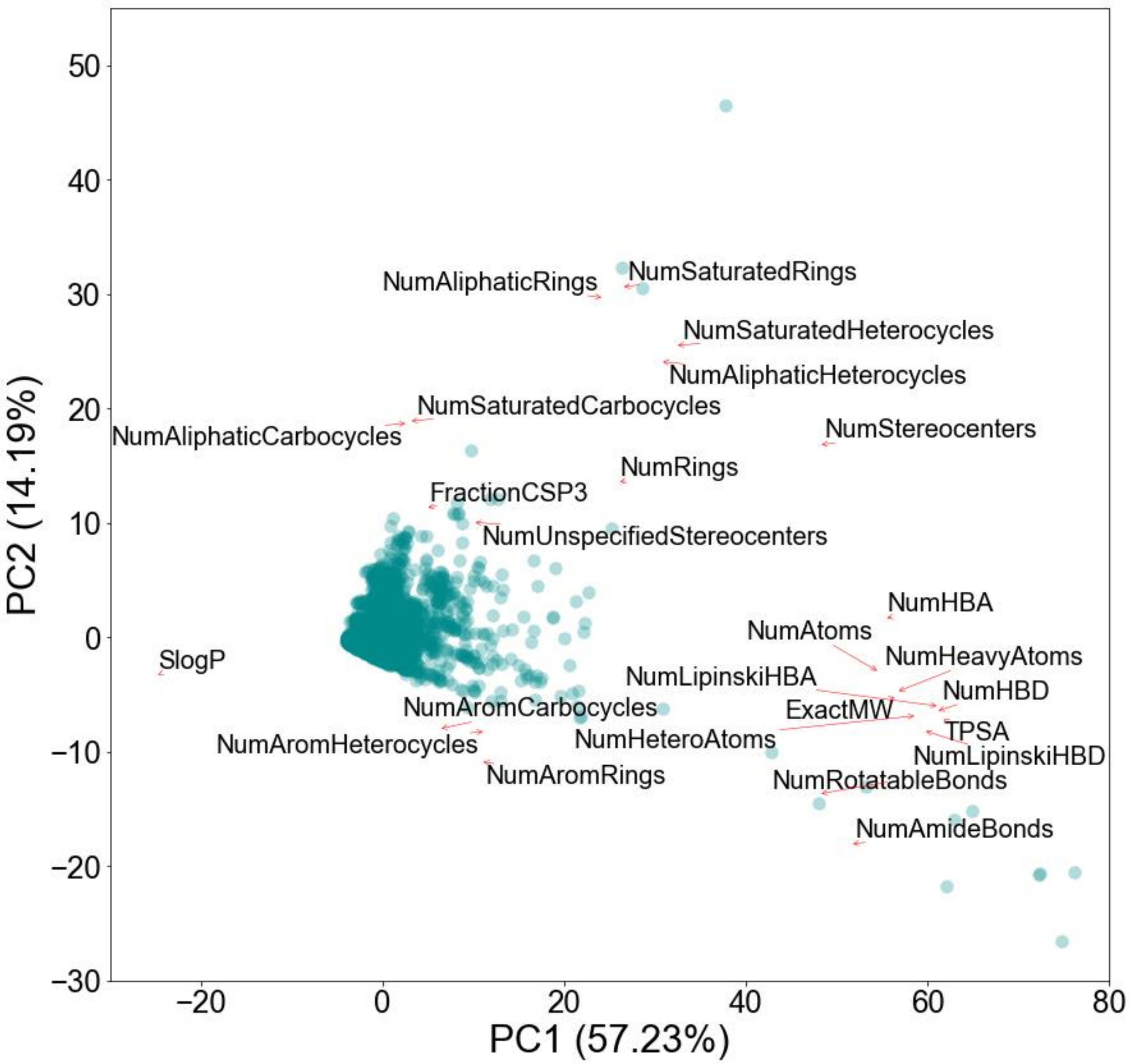

Figure S1. Loadings plot of the PCA based on a selection of interpretable molecular descriptors generated with RDKit on the global in vivo toxicity data set. The loadings plot shows how strongly each feature influences a principal component.

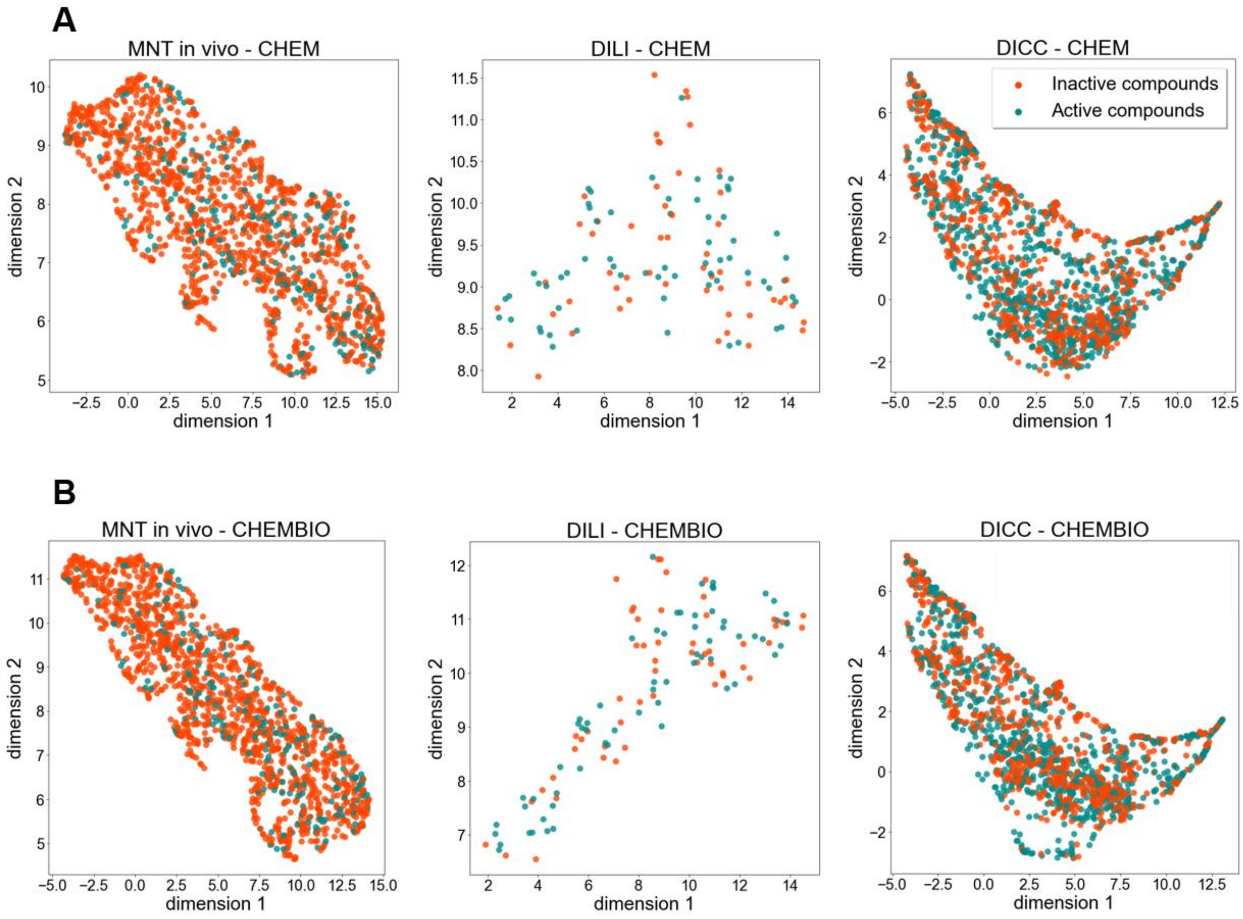

Figure S2. UMAP projections for the three in vivo endpoints (MNT in vivo, DILI and DICC) on (A) the CHEM descriptor set and (B) the CHEMBIO descriptor set.

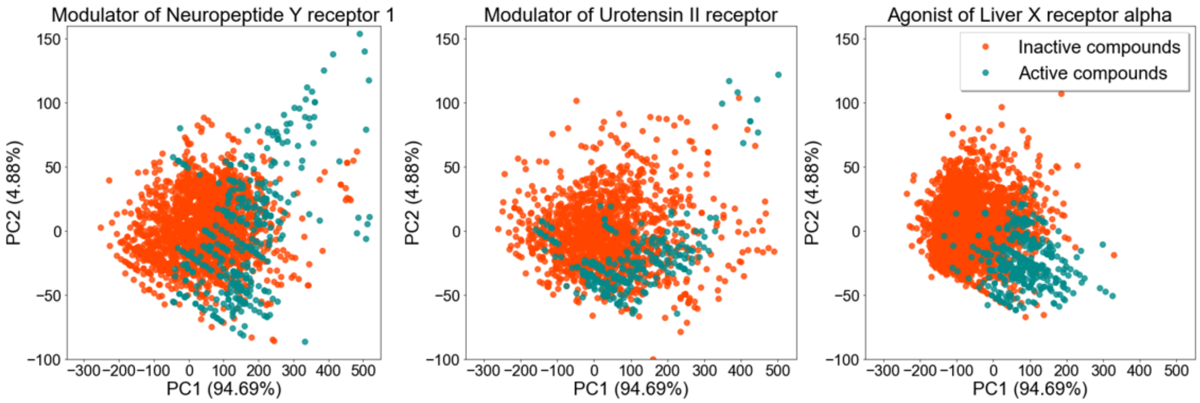

Figure S3. Principal component analysis based on a selection of interpretable molecular descriptors generated with RDKit. The PCA was derived from the merged data set of three eMolTox assays ("Modulator of Neuropeptide Y receptor type 1", "Modulator of Urotensin II receptor" and "Agonist of Liver X receptor alpha") for which the CP models yielded mean F1 scores on the single class predictions of 1.0. The active and inactive compounds of these data sets are located in differentiated parts of the chemical space, facilitating their classification.

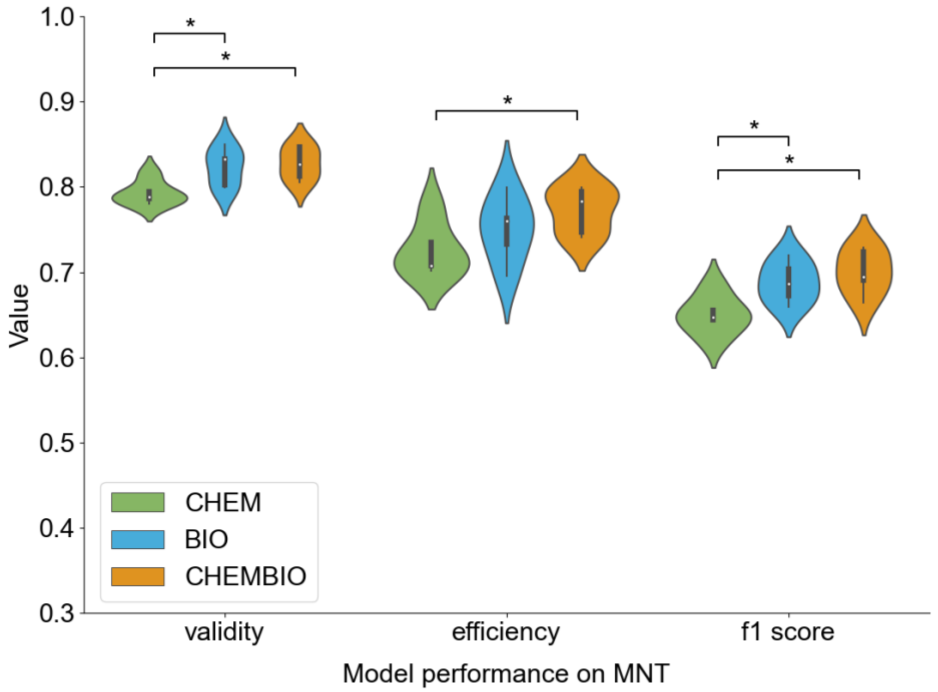
1.0
0.9
0.8
0.7
0.6
0.5
0.4
0.3
Value
*
*
*
*
*
CHEM
BIO
CHEMBIO
validity
efficiency
f1 score
Model performance on MNT

(a)

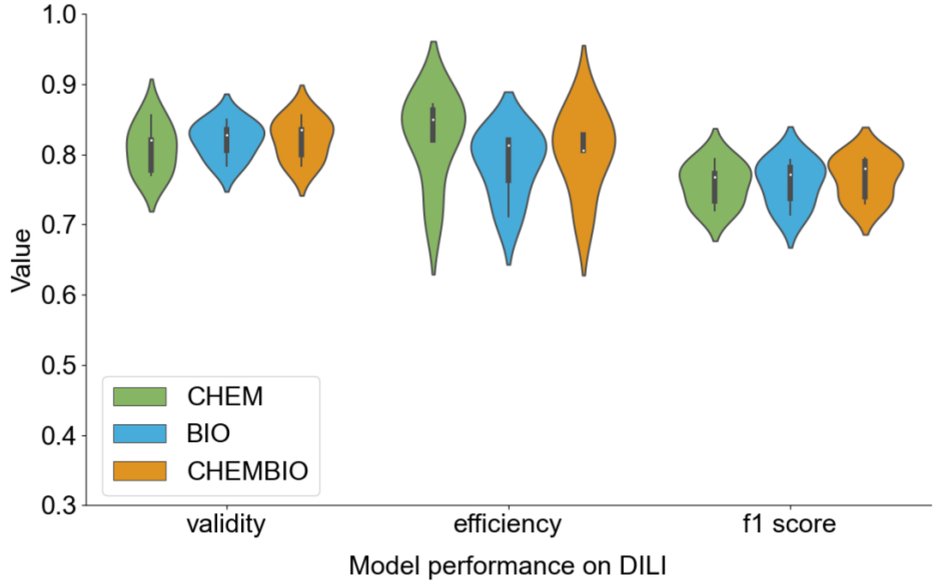
1.0
0.9
0.8
0.7
0.6
0.5
0.4
0.3
Value
CHEM
BIO
CHEMBIO
validity
efficiency
f1 score
Model performance on DILI

(b)

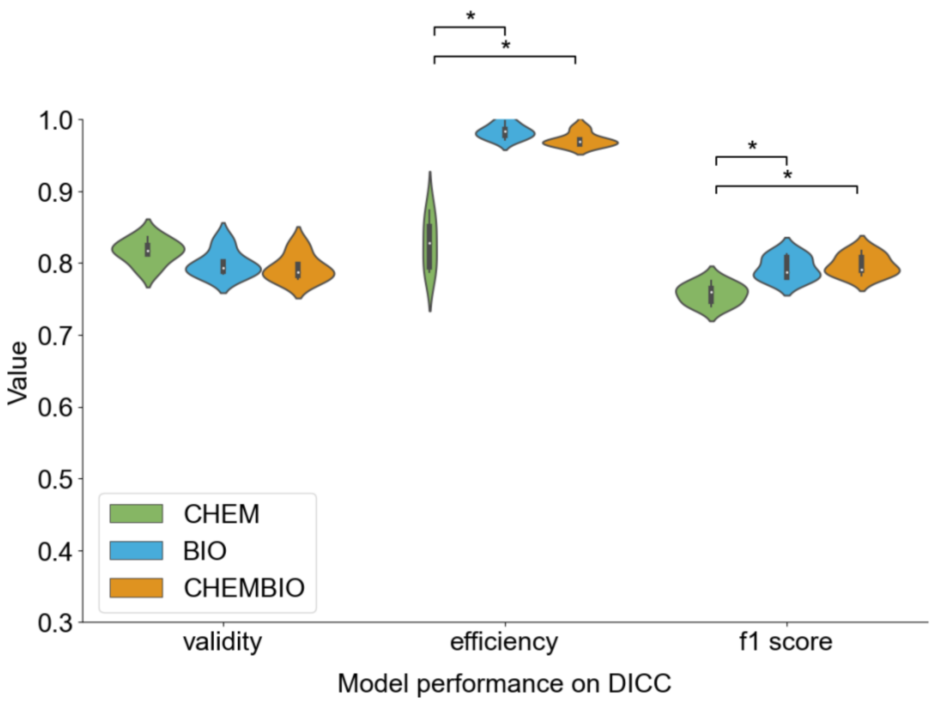

(c)

Figure S4. Distribution of the validity, efficiency and F1 score values obtained within the 5-fold CV framework for the (a) MNT, (b) DILI and (c) DICC CP models built on the different descriptor sets without feature selection. The CHEM descriptor set includes the molecular fingerprint and physicochemical descriptors; the BIO descriptor set includes the predicted p-values for a set of biological assays (bioactivity descriptor); the CHEMBIO descriptor set includes the previous two descriptor sets. Significant differences in the distribution (p-value < 0.05) are denoted by a star.

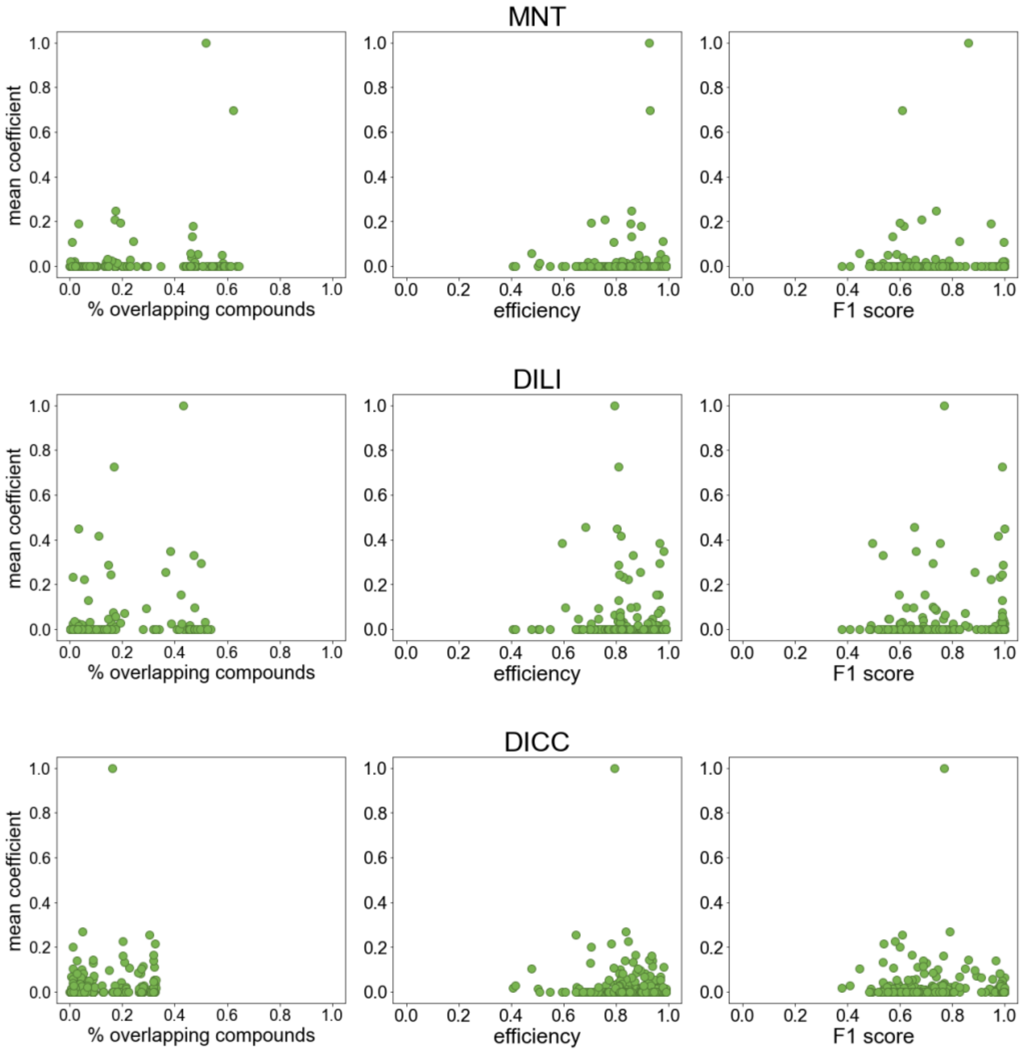

Figure S5. Mean coefficient reported by the lasso model for the bioactivity descriptors in relationship with the percentage of overlapping compounds (of the in vivo data set), the efficiency and F1 score of the models for each biological assay. For each of the 373 biological assays, the highest mean coefficient of the two p-values used as descriptors (for the active and inactive classes of each assay) was taken. The coefficients higher than 0 were normalized with a min-max normalization (from 0.01 to 1; see Materials and Methods section) for easier comparison.
